# Ultrasmall Ligand-Protected Ag_7_ Nanoclusters Enable Dual-Mode Reactive Oxygen Species Generation under Dark and Near-Infrared Irradiation

**DOI:** 10.1021/acsnano.6c01532

**Published:** 2026-05-28

**Authors:** Divinah Manoharan, Kana Yamamoto, Li-Chan Chang, Chouma Kurihashi, Issey Osaka, Yin-Fen Liu, Siou-Wei Liang, Hideya Kawasaki, Wen-Pin Su, Chen-Sheng Yeh

**Affiliations:** † Department of Chemistry, 34912 National Cheng Kung University, Tainan 701, Taiwan; ‡ Department of Chemistry and Materials Engineering, 12860 Kansai University, 3-3-35 Yamate-cho, Suita, Osaka 564-8680, Japan; § Institute of Clinical Medicine, College of Medicine, 34912 National Cheng Kung University, Tainan 704, Taiwan; ∥ Department of Pharmaceutical Engineering, Faculty of Engineering, 57948 Toyama Prefectural University, Imizu, Toyama 939-0398, Japan; ⊥ Center of Applied Nanomedicine, National Cheng Kung University, Tainan 701, Taiwan; # Departments of Oncology and Internal Medicine, National Cheng Kung University Hospital, College of Medicine, National Cheng Kung University, Tainan 704, Taiwan

**Keywords:** silver nanoclusters, self-oxygenation, ROS, photodynamic therapy, chemodynamic therapy

## Abstract

Ultrasmall silver nanoclusters, Ag_7_ NCs protected by MBISA (MBISA = 2-mercapto-5-benzimidazolesulfonic acid sodium salt), are presented as a dual-mode nanoplatform enabling complementary reactive oxygen species (ROS) activity under dark and near-infrared (NIR) light conditions. In darkness, Ag_7_ NCs catalyze hydrogen peroxide decomposition through electronically differentiated Ag sites, concurrently generating hydroxyl radicals (•OH) and molecular oxygen (O_2_). XPS analysis demonstrates that the electronically differentiated Ag sites are preserved after H_2_O_2_ exposure, indicating chemical robustness rather than sacrificial oxidation. Under 730 nm NIR irradiation, photoexcited Ag_7_ NCs transfer energy to the *in situ*-generated O_2_, yielding singlet oxygen (^1^O_2_). The H_2_O_2_-driven O_2_ supply can functionally support subsequent ^1^O_2_ generation under NIR irradiation. This cooperative mechanism enables stimulus-dependent activation of complementary ROS pathways for synergistic chemo- and photodynamic therapy. Ag_7_ NCs exhibit excellent stability, renal clearance, and biosafety, achieving potent tumor regression and metastasis suppression *in vivo*. These findings position Ag_7_ NCs as a molecularly defined nanocluster platform with redox-active behavior and complementary ROS for precision cancer therapy.

## Graphical abstract

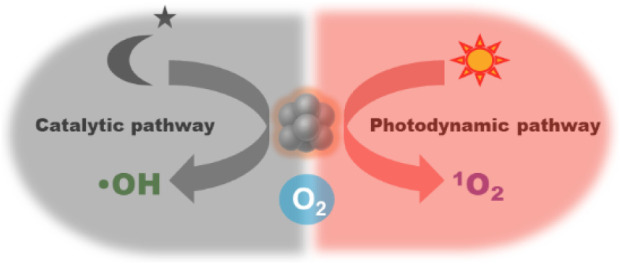

Atomically precise metal nanoclusters (NCs) have emerged as a distinctive class of ultrasmall materials positioned between molecular complexes and colloidal nanoparticles. Comprising only a few to several dozen atoms and typically <2 nm in diameter, NCs possess well-defined molecular formulas and discrete electronic states, giving rise to quantum confinement, tunable photophysics, and catalytic behaviors not attainable with larger nanostructures.^1,2^ Their favorable biocompatibility and renal-clearance profiles have prompted extensive exploration in bioimaging, biosensing, and cancer therapy.^3–5^

A central focus in nanomedicine is the ability to modulate reactive oxygen species (ROS)—key mediators of oxidative-stress-induced cell death. ROS such as singlet oxygen (^1^O_2_), hydroxyl radicals (•OH), and superoxide (O_2_•^–^) underpin photodynamic therapy (PDT), sonodynamic therapy (SDT), and chemodynamic therapy (CDT).^6–8^ Among these, PDT has attracted significant interest because ^1^O_2_ can be generated with high spatial selectivity upon light irradiation. Numerous nanomaterials, including gold NCs,^9^ copper-based catalysts,^10^ and porphyrinic systems,^11^ have been developed as photosensitizers to promote ^1^O_2_ formation under visible or near-infrared (NIR) excitation.

However, light-dependent therapeutic modalities face inherent limitations. Even NIR light penetrates only ∼1–2 cm into tissue, restricting efficacy in deep-seated tumors.^12^ Additionally, most nanomaterials operate via a single ROS pathway and cannot adapt to the heterogeneous microenvironments of solid tumors, where oxygen levels, H_2_O_2_ concentrations, and light accessibility vary dramatically.^13,14^ These challenges underscore the need for next-generation ROS platforms capable of (i) activating under NIR light to achieve improved penetration while also (ii) sustaining ROS production in darkness to extend therapeutic reach beyond the illuminated region.

Silver (Ag) NCs offer an attractive but underexplored opportunity for ROS engineering. Their high catalytic activity, ligand-dependent luminescence, and Fenton-like reactivity^15–18^ could, in principle, support versatile ROS modulation; however, photoinstability and oxidative degradation under physiological conditions have limited their translational potential.^19–21^ Robust and ultrasmall Ag NCs could therefore provide a powerful avenue for designing multimodal ROS nanoplatforms.

Here, we report the synthesis and functional characterization of ultrasmall silver nanoclusters with an Ag_7_-based core structure, stabilized by MBISA (MBISA = 2-mercapto-5-benzimidazolesulfonic acid sodium salt). These nanoclusters exhibit exceptional dispersibility, colloidal stability, and photochemical robustness under physiological conditions. For simplicity, these nanoclusters are hereafter referred to as Ag_7_ NCs. MBISA was selected because its sulfonate sodium group acts as a strong electrolyte with pH-independent dissociation, ensuring stable negative charge and excellent dispersion stability. Beyond earlier observations that Ag NCs can generate ^1^O_2_ under visible light,^22^ we demonstrate that Ag_7_ NCs exhibit a previously unrecognized dual-mode ROS activation via distinct, stimulus-dependent pathways operating in darkness and under NIR irradiation.

Under 730 nm NIR excitation, Ag_7_ NCs act as an efficient ^1^O_2_ sensitizer, enabling deep-tissue PDT. In darkness, the Ag_7_ NCs promote endogenous H_2_O_2_ activation via electronically differentiated Ag sites, yielding simultaneous generation of •OH and O_2_—an uncommon combination among ultrasmall NCs. This H_2_O_2_-driven oxygen generation can functionally support enhanced ^1^O_2_ production under NIR irradiation (Figure 1). Ag_7_ represents an ultrasmall Ag NC system exhibiting complementary ROS behavior in response to external stimuli under a “light-and-shadow” approach. Unlike conventional nanomaterials that rely solely on photodynamic mechanisms or complex composite architectures, Ag_7_ NCs enable complementary activation of oxidative stress under light and dark conditions. In light-accessible tumor regions, NIR-activated ^1^O_2_ formation drives photodynamic efficacy, whereas in deep, light-limited tissue zones, self-oxygenation-coupled •OH production ensures sustained cytotoxicity and continuous oxidative pressure.

**Figure 1:**
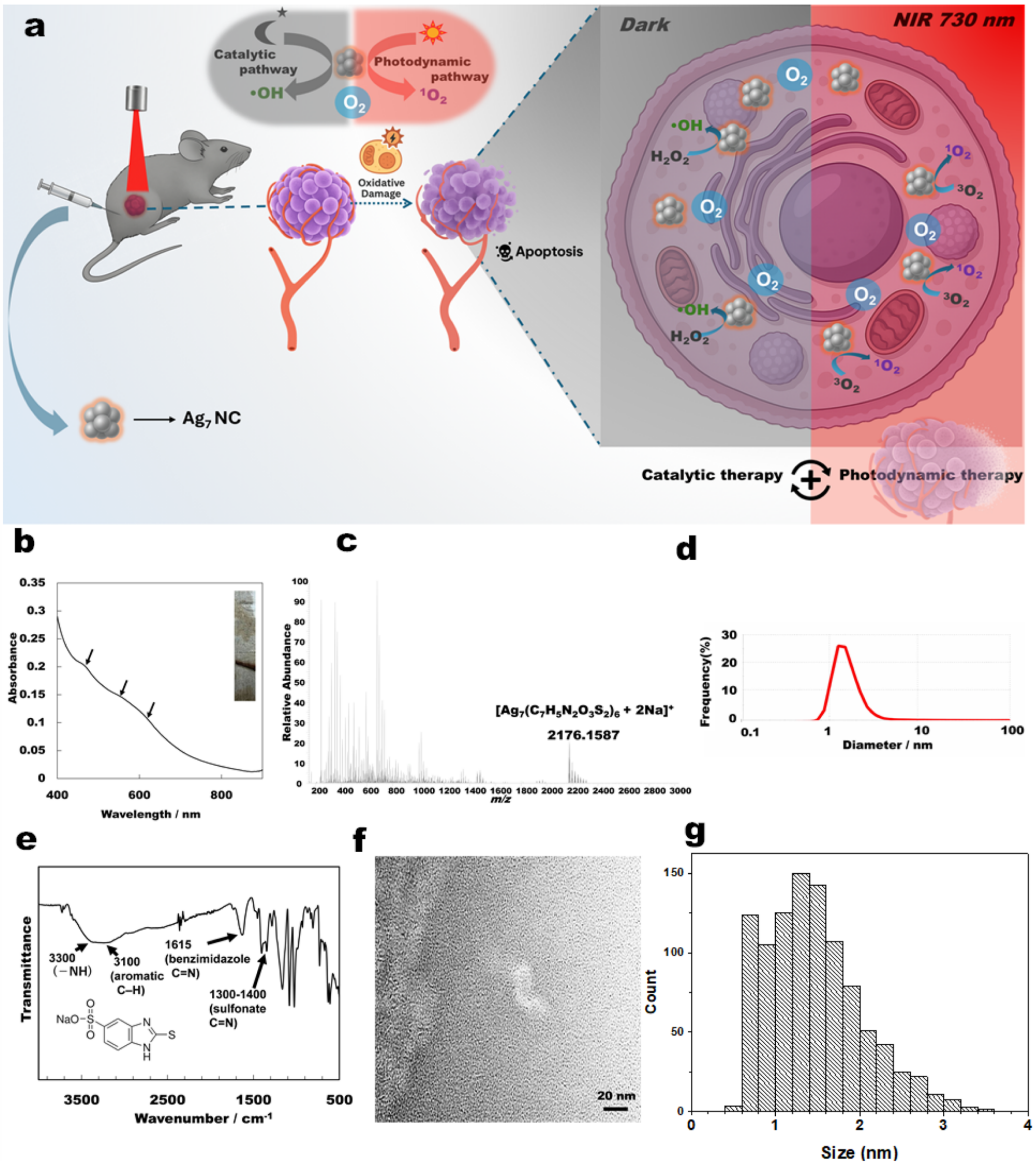
Structural and functional characterization of Ag_7_ NCs. (a) Schematic illustration of self-oxygenating Ag_7_ NCs-mediated cancer nanotherapy via orthogonal ROS pathways. Under NIR irradiation, Ag_7_ NCs produce singlet oxygen (^1^O_2_) for photodynamic therapy (PDT), while in dark condition, Ag_7_ NCs simultaneously generate O_2_ and hydroxyl radicals (•OH) through Fenton-like reactions for chemodynamic therapy (CDT), enabling synergistic and tumor-selective therapy. (Specific graphical elements namely the cell structure, tumor mass with blood vessel and mice in panel (a) were created with ChatGPT Image 2.0, while other elements were created manually using Microsoft PowerPoint and Paint. The elements were subsequently compiled, modified, assembled and finalized using Microsoft PowerPoint.) (b) UV–vis absorption spectrum of Ag_7_ NCs showing discrete molecular-like features with peaks at ∼470, 565, and 605 nm. Inset: PAGE image exhibiting a single, sharp band, indicating monodispersity. (c) ESI-MS spectrum showing the dominant *m*/*z* peak 2176.1587 of Ag_7_(MBISA)_6_ NCs. (d) Size distribution of Ag_7_ NCs measured by DLS, showing an average hydrodynamic diameter of ca. 1.3 nm. (e) FT-IR spectrum of Ag_7_ NCs confirming MBISA surface functionalization. The absence of the free S–H band and presence of characteristic bands for −NH, aromatic C–H, C=N, and C–S stretching confirms ligand coordination. (f) Transmission electron microscopy (TEM) images of Ag_7_ NCs showing uniformly dispersed dark contrast spots corresponding to individual NCs. (g) The corresponding size distribution histogram obtained from *n* = 1000 NCs reveals an average diameter of 1.19 ± 0.73 nm, with the majority of clusters falling within the 0.7–2 nm range.

Collectively, these discoveries establish Ag_7_ NCs as a prototype “light-and-shadow” nanoplatform that integrates photodynamic and chemodynamic functions within a single molecularly defined structure. In a stimulus-responsive manner, the system enables modulation of oxidative stress, indicating its potential relevance for precision cancer therapy.

## Results and Discussion

### Preparation and Characterization of Ag_7_ NCs

The structural and photophysical properties of Ag_7_ NCs were thoroughly investigated to evaluate their potential as photoactive therapeutic agents. As shown in Figure 1b, the UV–vis absorption spectrum exhibited distinct molecular-like features with characteristic peaks centered at approximately 470, 565, and 605 nm, indicative of discrete electronic transitions. Polyacrylamide gel electrophoresis (PAGE) analysis further confirmed the presence of a uniform population of ultrasmall nanoclusters, showing a single, sharp band with no evidence of aggregation or degradation (inset, Figure 1b). ESI–MS analysis identified a dominant peak at *m*/*z* = 2176.1587 (Figure 1c), which is consistent with a composition assignable to Ag_7_(MBISA)_x_ (x ≈ 6), although the exact ligand stoichiometry is not strictly determined. The observed isotopic distribution matches well with the simulated pattern of the Ag_7_ NCs (Figure S1), supporting the formation of ultrasmall Ag_7_-based nanoclusters stabilized by MBISA ligands. Additional discussion of the fragmentation behavior and minor peaks is provided in the Supporting Information. Although the ESI–MS and isotopic pattern analyses support the formation of Ag_7_ NCs, the precise atomic-level structure, including the Ag-core arrangement and MBISA ligand coordination mode, has not yet been determined. Single-crystal X-ray diffraction analysis will be necessary to clarify these structural details in future work. Dynamic light scattering (DLS) analysis revealed that the Ag_7_ NCs have an average hydrodynamic diameter of about 1.3 nm, consistent with their ultrasmall size (Figure 1d). Surface functionalization with MBISA was confirmed by FT-IR spectroscopy (Figure 1e). The absence of a free S–H stretching band in the 2000–2500 cm^–1^ region indicated successful thiol binding to the silver core. Additional characteristic bands were observed at 3300 cm^–1^ (−NH stretching), 3100 cm^–1^ (aromatic C–H stretching), 1615 cm^–1^ (C=N stretching of the benzimidazole ring), and 1300–1400 cm^–1^ (C–S stretching of the sulfonate group). In the S 2p XPS spectra (Figure S2), two components were observed at binding energies of 162.1 and 167.9 eV. The lower-binding-energy peak at 162.1 eV is attributed to thiolate-type Ag–S bonding, consistent with reported values for metal–sulfur coordination.^23^ The higher-binding-energy component at 167.9 eV corresponds to fully oxidized sulfur species, in agreement with the sulfonate functionality of the MBISA ligand. Zeta potential measurements confirmed that the surface of the Ag_7_ NCs was negatively charged due to the presence of sulfonate groups, with a measured value of −29 mV.

Figure 1f shows a representative TEM image of Ag_7_ NCs, supporting their ultrasmall size and good dispersion. Size analysis from multiple TEM images yielded the distribution shown in Figure 1g, with most clusters falling within the sub-2 nm regime. The mean diameter was 1.19 ± 0.73 nm. For ultrasmall clusters, the apparent size distribution obtained from TEM images can appear broadened due to the limited spatial resolution of TEM and the influence of the surrounding ligand shell. In particular, the observed size reflects the overall cluster including ligand contributions and thus overestimates the metallic core size. Overall, the TEM results support the formation of ultrasmall Ag NCs in the sub-2 nm regime, consistent with a predominantly uniform nanocluster population in solution. At higher electron beam intensities, NCs exhibited aggregation and coalescence into larger nanoparticles, a phenomenon commonly observed in ultrasmall metal clusters.^24^ High-resolution TEM revealed well-dispersed spherical nanoparticles with distinct lattice fringes (Figure S3a,b), confirming their crystalline nature. The corresponding SAED pattern displayed indexed diffraction rings characteristic of metallic silver’s face-centered cubic (fcc) structure (Figure S3c). The size distribution derived from multiple HRTEM images (Figure S3d) indicated a population of nanoparticles with an average diameter of 3.8 ± 0.5 nm, reflecting beam-induced growth during imaging rather than the intrinsic size of the Ag_7_ NCs.

### The Photosensitizing Ability of the Ag_7_ NCs

The photosensitizing ability of the Ag_7_ NCs was assessed using the 9,10-anthracenediyl-bis­(methylene)­dimalonic acid (ABDA) assay under NIR irradiation (730 nm, 26 mW cm^–2^). The irradiation wavelength of 730 nm was chosen because it lies within the NIR-I biological window, where hemoglobin absorption is reduced and water absorption remains low, allowing effective tissue penetration. Although 808 nm is often used for NIR excitation, Ag_7_ shows negligible absorption and no detectable singlet oxygen generation at 808 nm, whereas 730 nm overlaps with its long-wavelength absorption tail and enables effective photosensitization.

In D_2_O, ABDA absorbance at 379 nm decreased markedly (*k* = 7.2 × 10^–2^ min^–1^), indicating efficient generation of ^1^O_2_ (Figure 2a). Electron spin resonance (ESR) spectroscopy using TEMP as a ^1^O_2_ probe further supported this result, showing a much stronger TEMPO signal under 730 nm irradiation in the presence of Ag_7_ NCs than in the control (Figure 1b). The ^1^O_2_ quantum yield (Φ) of Ag_7_ NCs was determined to be 8.4% under 730 nm irradiation using Indocyanine Green (ICG) as a reference standard based on the ABDA bleaching method,^25^ corresponding to a 42-fold enhancement relative to ICG (Figure S4). This value demonstrates the superior ^1^O_2_ sensitization capability of Ag_7_ NCs compared with this clinically established NIR photosensitizer.

**Figure 2:**
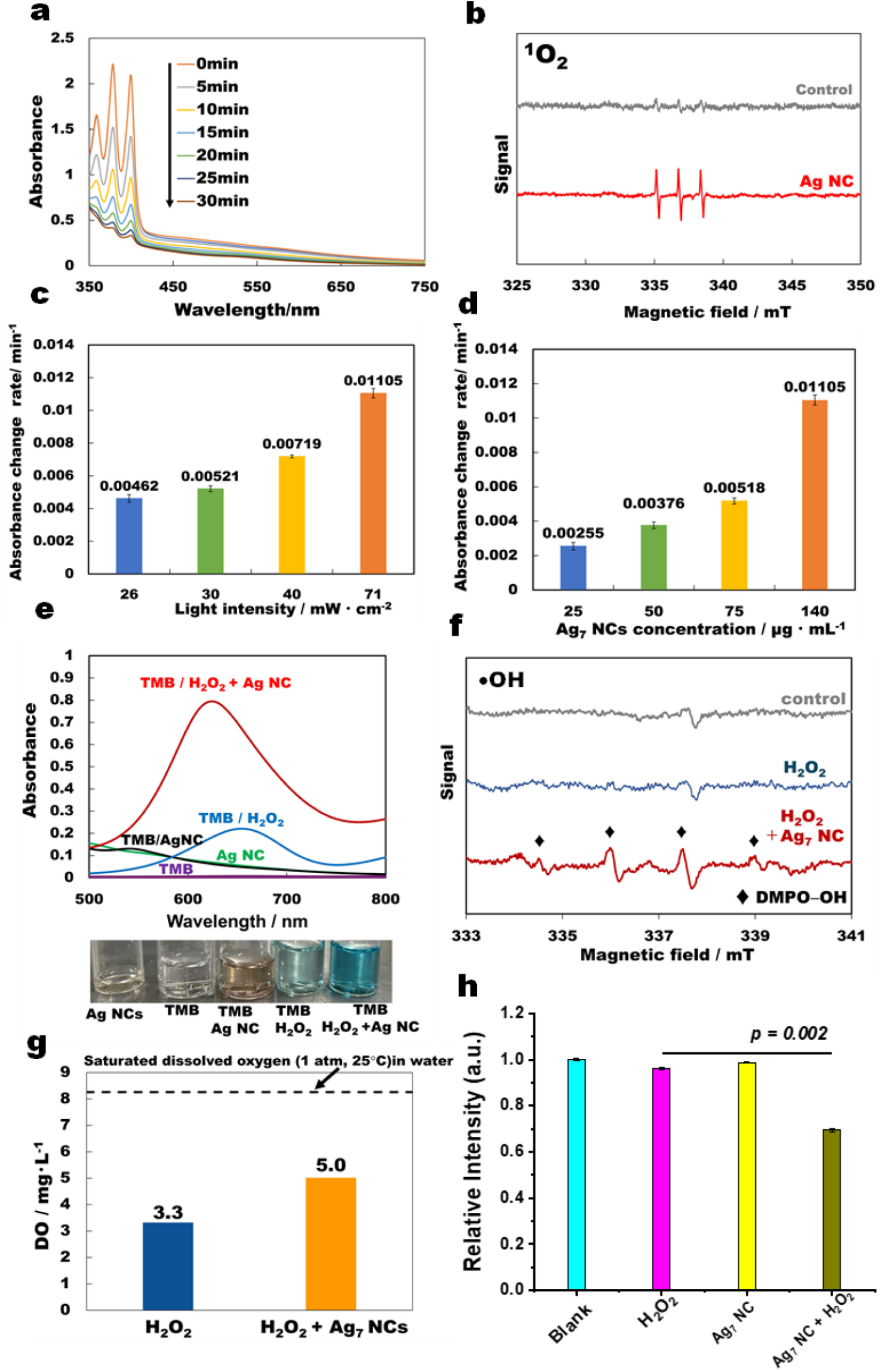
Orthogonal ROS generation pathways of Ag_7_ NCs under light and dark conditions. (a) ABDA degradation under NIR irradiation indicates efficient ^1^O_2_ production. (b) ESR spectrum using TEMP showing light-activated ^1^O_2_ generation. (c), (d) Positive correlation of ^1^O_2_ generation rate with Ag_7_ NCs concentration and irradiation intensity. (e) TMB assay revealing dark-activated peroxidase-like activity of Ag_7_ NCs in the presence of H_2_O_2_. (f) ESR spectrum with DMPO showing •OH generation under dark conditions with H_2_O. (g) Dissolved O_2_ measurements demonstrating catalytic O_2_ production from H_2_O_2_. (h) O_2_ generation by Ag_7_ NCs (10 ppm) in the presence of H_2_O_2_ (10 μM) using [Ru­(dpp)­3]­Cl_2_ (ruthenium­(II) tris­(4,7-diphenyl-1,10-phenanthroline) dichloride) as the oxygen-sensitive probe in PBS 6.4 (purged with N_2_), with reaction time of 10 min using λ_ex_ = 450 nm and λ_em_ = 615 nm. (*n* = 3). Data are mean ± s.e.m.

When D_2_O was replaced with H_2_O, the degradation rate decreased to *k* = 4.6 × 10^–3^ min^–1^ (approximately a 15-fold reduction). This clear isotope effect, due to the longer lifetime of ^1^O_2_ in D_2_O, supports the role of singlet oxygen in reactive oxygen species (ROS) generation. In photosensitized systems, ^1^O_2_ can form either through electron-transfer pathways involving superoxide intermediates or through energy transfer from an excited photosensitizer to ground-state oxygen. The observed isotope effect indicates that the energy-transfer (Type II) pathway is the main mechanism in this system. In addition, ^1^O_2_ production increased with both Ag_7_ NC concentration and light intensity (Figure 2c,d), confirming that photoexcited Ag_7_ NCs drive ROS generation.

To examine the contribution of electron-transfer pathways, we introduced sodium 4,5-dihydroxybenzene-1,3-disulfonate (5 mM), a common superoxide scavenger, under the same NIR irradiation conditions.^26^ Because O_2_•^–^ can participate in Type I processes that lead to secondary ^1^O_2_ formation, its removal should affect the outcome if this pathway is significant. However, the ABDA decay profile remained nearly unchanged (Figure S5), and the rate constant decreased only slightly from 7.2 × 10^–2^ to 6.4 × 10^–2^ min^–1^ (approximately 11% reduction). This result suggests that O_2_•^–^-mediated pathways play only a minor role under the present conditions. Taken together, these results show that energy transfer (Type II) is the dominant pathway for ^1^O_2_ generation, while electron-transfer processes contribute only secondarily. Precise quantification of each contribution is difficult; therefore, this conclusion is based on relative comparisons.

### Colloidal Stability of the Ag_7_ NCs

The Ag_7_ NCs exhibited excellent stability under various physiologically relevant conditions. Over a period of 5 days, the NCs remained well-dispersed in deionized distilled water, phosphate-buffered saline (PBS, pH 7.0 and 6.0), and Dulbecco’s Modified Eagle Medium (DMEM), with no visible signs of aggregation or precipitation (Figure S6a). Dynamic light scattering (DLS) measurements showed that Ag_7_ NCs maintained a nearly constant hydrodynamic diameter (Figure S6b) over 5 days in biologically relevant media, indicating good colloidal stability with no detectable aggregation. This stability is attributable to the strongly hydrophilic, anionic MBISA ligand shell. High-resolution TEM images taken on day 0 and day 5 revealed preserved morphology and crystalline lattice features, as confirmed by SAED patterns and fast Fourier transform (FFT) analysis (Figure S6c). Furthermore, a negligible increase in temperature (less than 2 °C) was observed in PBS containing 100 ppm Ag_7_ NCs during continuous NIR irradiation (730 nm, 71 mW cm^–2^) (*n* = 3), without any degradation (Figure S6d). Photostability was also demonstrated under continuous NIR irradiation (730 nm, 71 mW cm^–2^). UV–vis absorption spectra recorded every 5 min during 30 min irradiation showed negligible shifts in peak position or intensity, confirming that the optical properties of Ag_7_ NCs remained unchanged (Figure S6e). The time-dependent release of dialyzable silver species from MBISA-functionalized Ag_7_ NCs was evaluated under dark physiological buffer conditions using dialysis-assisted ICP-MS as shown in Figure S6f. Blank HEPES controls showed no detectable Ag signal. There is no dialyzable silver released (<0.015%) in HEPES. This result indicates that the MBISA-functionalized Ag_7_ NCs possess high chemical stability under dark physiological conditions and do not undergo rapid Ag dissociation. This proves that their biological activity is unlikely to arise from uncontrolled silver ion leaching.

### ROS Generation and Self-Oxygenation in Dark Conditions

Nanozymes—nanomaterials that emulate enzymatic functions while offering superior stability, tunable reactivity, and low-cost production—have emerged as promising catalysts for chemodynamic therapy (CDT).^27^ In particular, nanozymes with peroxidase- or Fenton-like activity can convert endogenous hydrogen peroxide (H_2_O_2_) into highly cytotoxic hydroxyl radicals (•OH). Ag_7_ NCs display such activity even in the absence of light. A substantial increase in the TMB absorbance at 650 nm was observed only when both Ag_7_ NCs and H_2_O_2_ were present Figure 2e), and ESR analysis with DMPO revealed a characteristic DMPO–OH adduct signal (Figure 2f), confirming •OH generation. Absolute quantification of •OH was not attempted due to its extremely short lifetime and the lack of stable reference standards with defined •OH concentrations for direct calibration; however, its generation was consistently confirmed by ESR spin-trapping and probe-based assays.^28–30^

Beyond •OH production, Ag_7_ NCs catalytically accelerate the decomposition of H_2_O_2_ into molecular oxygen. Despite prior deaeration, solutions containing Ag_7_ NCs exhibited higher dissolved O_2_ levels than H_2_O_2_ alone, underscoring their catalytic contribution to O_2_ evolution (Figure 2g). This H_2_O_2_-driven oxygen supply is particularly advantageous for locally increasing O_2_ availability to overcome a key limitation of photodynamic processes and potentiate the NIR-induced formation of ^1^O_2_. Figure 2h demonstrates the oxygen generation capability of Ag_7_ NCs in the presence of H_2_O_2_, at pH 6.4. Neither H_2_O_2_ (10 μM) nor Ag_7_ NCs (10 ppm) alone quenched the Ru­(dpp)_3_ red emission after 10 min, whereas Ag_7_ NCs in the presence of H_2_O_2_ caused a clear reduction in intensity, indicating O_2_ generation that quenches the Ru­(dpp)_3_ red emission. To further examine whether oxygen generated from H_2_O_2_ decomposition contributes to enhanced photodynamic activity, ^1^O_2_ generation was evaluated using ABDA as a chemical probe. Under identical 730 nm irradiation conditions, the degradation rate of ABDA was significantly higher for Ag_7_ NCs in the presence of Ag NCs + H_2_O_2_ system than for Ag_7_ NCs alone or H_2_O_2_ alone, supporting that *in situ* oxygen generation enhances NIR-induced ^1^O_2_ production (Figure S7).

Ag NCs are often reported to undergo oxidation in the presence of H_2_O_2_ because hydrogen peroxide can modify the electronic state of surface Ag atoms.^31^ To examine whether such changes occur in the present system, the electronic structure of Ag atoms in Ag_7_ nanoclusters was analyzed by XPS. The Ag 3d_5/2_ spectrum can be deconvoluted into two components located at 368.1 and 367.3 eV (Figure S8). Rather than assigning these peaks to discrete Ag­(I) and Ag(0) oxidation states—which can be ambiguous for ultrasmall metal nanoclusters due to final-state effects and size-dependent electronic screening—the two components are interpreted as reflecting electronically differentiated Ag environments within the nanocluster structure. The dominant higher-binding-energy component (368.1 eV, ≈95%) is associated with ligand-coordinated surface Ag environments, whereas the minor lower-binding-energy component (367.3 eV, ≈5%) corresponds to relatively core-like Ag environments, consistent with site-dependent electronic structures reported for thiolate-protected silver nanoclusters.^32^

Quantitative analysis of the XPS peak areas shows that the lower- and higher-binding-energy components account for 5.3% and 94.7%, respectively, before H_2_O_2_ exposure, and remain essentially unchanged (4.5% and 95.5%) after treatment with 10 mM H_2_O_2_ under dark conditions (Figure S8). Consistently, the overall Ag MNN Auger spectral profile also remains essentially unchanged after H_2_O_2_ exposure (Figure S9). These combined results indicate that the overall electronic-state distribution of Ag atoms in the Ag_7_ NCs is largely preserved even in the presence of H_2_O_2_, suggesting that the nanocluster framework remains electronically stable during the catalytic activation of H_2_O_2_.

Combined with the concurrent generation of •OH and O_2_ observed in the Ag_7_-based reaction system, these results suggest that H_2_O_2_ activation occurs at electronically heterogeneous Ag sites within the nanocluster rather than through irreversible oxidation of the cluster framework. Such electronic heterogeneity can influence the adsorption and activation of H_2_O_2_ at the cluster surface, allowing multiple catalytic decomposition pathways to operate. In this scenario, O–O bond cleavage of adsorbed H_2_O_2_ can generate •OH radicals, whereas parallel pathways may contribute to O_2_ formation. These findings highlight the importance of site-specific electronic heterogeneity in ultrasmall metal nanoclusters for catalytic H_2_O_2_ activation and the generation of reactive oxygen species.

The tumors characterized by elevated H_2_O_2_ levels and heterogeneous microenvironment^33^ provide a biochemical niche that Ag_7_ NCs can effectively exploit. The NCs generate •OH *in situ* without external activation, enabling CDT, while simultaneously enhance photoinduced ^1^O_2_ production. Consequently, Ag_7_ NCs function as dual-mode therapeutic agents, producing •OH in the dark and ^1^O_2_ under NIR irradiation, offering a synergistic pathway for potentiated cancer therapy.

### Time-Dependent Cell-Associated Ag_7_ NCs

The time-dependent cell-associated Ag originating from Ag_7_ NCs in MDA-MB-231 breast cancer cells was quantified using ICP-MS (Figure 3a). ICP-MS quantifies total cell-associated Ag and does not strictly discriminate residual surface-bound from internalized fractions. The cell-associated Ag increased steadily with incubation time, showing a sharp rise within the first 6 h and approaching a plateau by 24 h, consistent with progressive cellular association. Based on this kinetic profile, 24 h was selected for subsequent studies to ensure robust cellular association of Ag_7_ NCs prior to therapeutic activation.

**Figure 3:**
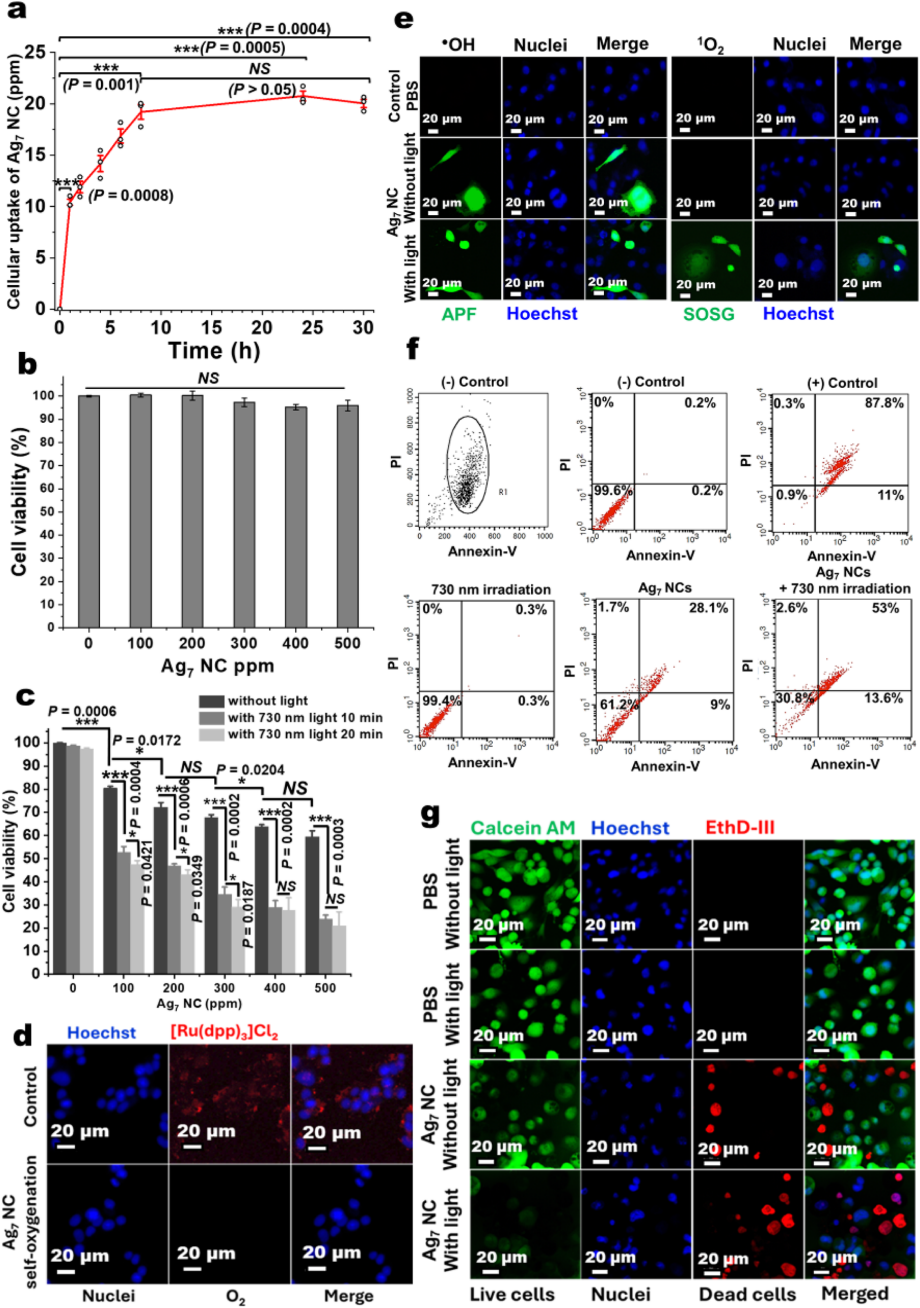
*In vitro* evaluation of Ag_7_ NCs for cancer nanotherapy. (a) Time-dependent uptake behavior of Ag_7_ NCs in MDA-MB-231 breast cancer cell line (*n* = 3). Data are mean ± s.e.m. (b) Concentration-dependent cytotoxicity of Ag_7_ NCs incubated with normal epithelial cell line MCF-10A for 24 h in dark condition evaluated by MTT assay (*n* = 3). Data are mean ± s.e.m. (c) Concentration-dependent cytotoxicity of Ag_7_ NCs incubated with breast cancer cell line MDA-MB-231 for 24 h and subsequent exposure to 730 nm irradiation for 0, 10, and 20 min evaluated by MTT assay (*n* = 3). (d) Confocal fluorescence images demonstrating intracellular oxygen elevation in cancer cells after treatment with Ag_7_ NCs for 24 h. The oxygen-sensitive probe [Ru­(dpp)_3_]­Cl_2_ (tris­(4,7-diphenyl-1,10-phenanthroline)­ruthenium­(II) chloride) exhibits red phosphorescence that is quenched in the presence of oxygen. Compared to the control group (cancer cells without treatment), Ag_7_-treated cells display no red emission, indicating catalytic decomposition of intracellular H_2_O_2_ into O_2_ and self-oxygenation. Scale bar: 20 μm. (e) Representative fluorescence images of MDA-MB-231 cells after treatment with Ag_7_ NCs showing intracellular ROS generation: light-independent •OH levels and light-dependent ^1^O_2_ production following 730 nm irradiation for 10 min. The •OH radical was detected using APF (green) and ^1^O_2_ was detected using SOSG (green) in two separate staining experiments, with nuclei counterstained by Hoechst 33342 (blue). Images are the representative of three independent experiments. Scale bars, 20 μm. (f) Flow cytometry analysis of MDA-MB-231 cells untreated (negative control), treated with a potent cytotoxin thapsigargin to induce apoptosis (positive control), treated with 730 nm irradiation only for 20 min without Ag_7_ NCs and treated with 300 ppm Ag_7_ NCs with/without 730 nm irradiation for 20 min. (g) Representative fluorescence images of the distribution of MDA-MB-231 live and dead cells untreated, treated with 730 nm light only, and treated with 300 ppm Ag_7_ NCs with/without 730 nm irradiation. The nuclei, live cells and dead cells were stained by Hoechst 33342 (blue), calcein AM (green) and ethidium homodimer-III (EthD-III, red), respectively. Images are the representative of three independent experiments. Scale bars, 20 μm.

### Cytotoxicity toward Normal and Cancer Cells in Dark and Light Conditions

The cytocompatibility of Ag_7_ NCs was first evaluated in normal breast epithelial cells (MCF-10A) using the MTT assay. No significant cytotoxicity was observed at the highest tested concentration (500 ppm) (Figure 3b), indicating negligible off-target toxicity. This observation was further supported by live/dead cell staining, which demonstrated that MCF-10A cells retained intact membrane integrity and nuclear morphology after 24 and 48 h incubation with Ag_7_ NCs (Figure S10). The absence of EthD-III uptake confirmed cytocompatibility in normal cells. This proves that the Ag_7_ NCs are nontoxic to normal cells under dark condition. In contrast, MDA-MB-231 breast cancer cells exhibited clear concentration- and light-dependent cytotoxicity (Figure 3c). Without irradiation, the Ag_7_ NCs induced a modest decrease in viability, consistent with basal •OH production in dark condition. With the highest concentration of 500 ppm under dark conditions, the cancer cell viability dropped to 60%, compared to 95% in normal cells. This selectivity is based on overexpressed endogenous H_2_O_2_ in tumor cells. However, upon 730 nm irradiation (71 mW cm^–2^), cell death increased significantly in both a dose- and irradiation time-dependent manner. The half-maximal inhibitory concentration (IC_50_) was determined to be 100 ppm with 20 min irradiation and 200 ppm with 10 min irradiation. The highest inhibitory effect was observed for higher concentration of 500 ppm with 20 min irradiation as the viability dropped to ∼20%. A dose of 300 ppm of Ag_7_ NCs with 20 min irradiation can decrease the viability to 30%. These findings confirm that photodynamically generated ^1^O_2_ substantially enhances the therapeutic response of Ag_7_ NCs. For subsequent mechanistic and therapeutic studies, 300 ppm with 20 min irradiation was selected as an optimal working condition, balancing efficacy with minimized dosage.

### Intracellular Self-Oxygenating and ROS Generation in Dark

We next assessed intracellular oxygenation using the dissolved-oxygen probe [Ru­(dpp)_3_]­Cl_2_ in cancer cells. As shown in Figure 3d, Ag_7_ NCs-treated cells exhibit a marked quenching of red phosphorescence relative to untreated controls (nuclei counterstained with Hoechst (blue)). This is because of the sensor quenching by elevated intracellular O_2_ produced from endogenous H_2_O_2_. This cellular readout aligns with the tube assay trend in Figure 2h, where only the Ag_7_ NCs + H_2_O_2_ condition reduced Ru­(dpp)_3_ emission after 10 min in N_2_-purged PBS buffer (pH 6.4). Together, these results indicate that Ag_7_ NCs can self-oxygenate the local microenvironment in cells without light input.

Fluorescence staining provided evidence supporting complementary ROS activation under dark vs NIR conditions. Aminophenyl fluorescein (APF), an •OH probe, showed strong intracellular green fluorescence even without irradiation, confirming that Ag_7_ NCs catalyze pseudo-Fenton-like reactions in the presence of endogenous H_2_O_2_ (Figure 3e). In contrast, Singlet Oxygen Sensor Green (SOSG), a singlet oxygen probe, exhibited a strong response after 730 nm irradiation, confirming efficient photodynamic ^1^O_2_ generation. These complementary pathways validate the unique “light-and-shadow” therapeutic mechanism of Ag_7_ NCs, where •OH dominates in dark tumor environments and ^1^O_2_ is activated under NIR light. It should be noted that independent and fully quantitative discrimination of multiple ROS species in complex biological environments remains challenging; therefore, probe-based assays are interpreted as semiquantitative indicators of relative trends.

### Mechanism of Cell Death

Flow cytometry (Annexin V/PI staining) showed that Ag_7_ NCs alone induced ∼37% apoptosis, attributable to •OH generation (Figure 3f). With 730 nm irradiation, the apoptotic fraction increased to ∼67%, reflecting the synergistic effect of dual ROS production. The 730 nm light alone group did not cause cell death and is similar to the negative untreated control group. For comparison, a positive control group with cells treated by a potent cytotoxin, thapsigargin, to induce apoptosis is included, which shows a ∼99% cell apoptosis. The flow cytometry results are consistent with the MTT assay results.

### Live/Dead Staining in Dark and Light Conditions

Confocal imaging further confirmed this therapeutic outcome (Figure 3g). The nuclei, live cells, and dead cells were stained by Hoechst 33342 (blue), calcein AM (green), and ethidium homodimer-III (red), respectively, for qualitative imaging evaluation. PBS or light alone had negligible effects. Ag_7_ NCs in the dark induced partial cell death (∼32%), while Ag_7_ NCs with 730 nm irradiation resulted in extensive membrane damage and nuclear fragmentation, leading to cell death (∼71%), with strong EthD-III uptake and loss of calcein AM fluorescence.

### Hemocompatibility of Ag_7_ NCs

The hemocompatibility of Ag_7_ NCs was assessed using a standard RBC hemolysis assay with PBS and deionized water as the 0% and 100% hemolysis controls, respectively. Under the tested conditions, Ag_7_ NC induced negligible hemolysis (≈1%) as shown in Figure S11, indicating minimal disruption of erythrocyte membranes. Importantly, the hemoglobin readout at OD_540nm_ was background-corrected by subtracting the absorbance of Ag_7_ NCs dispersed in PBS without RBCs, excluding spectral overlap from the nanoclusters and supporting the robustness of the quantification. The low hemolytic activity is consistent with the ultrasmall size and strong ligand protection of Ag_7_ NCs. TEM revealed a sub-2 nm core, while DLS showed a small hydrodynamic diameter (∼2–3 nm) indicative of a compact ligand/hydration shell. The MBISA coating is highly hydrophilic and anionic (sulfonate-containing), which is expected to reduce nonspecific adsorption to the negatively charged RBC surface and limit direct lipid–metal interactions that can trigger membrane destabilization. Together with the observed colloidal stability across physiologically relevant media, these results suggest that Ag_7_ NCs maintains a well-dispersed, passivated state that mitigates acute hemotoxicity, supporting its suitability for subsequent biological and therapeutic evaluations.

### In Vivo Evaluation of Ag_7_ NCs for Cancer Nanotherapy

After confirming the *in vitro* cytotoxicity of Ag_7_ NCs, demonstrating selective activity against MDA-MB-231 breast cancer cells, and their synergistic effect under 730 nm irradiation, we further investigated the *in vivo* antitumor efficacy of Ag_7_ NCs. Given its ultrasmall size (<2 nm), Ag_7_ NCs would likely undergo rapid renal clearance if administered systemically via conventional tail-vein injection, thereby preventing effective tumor accumulation through the enhanced permeability and retention (EPR) effect. Accordingly, we adopted an intratumoral injection strategy for drug delivery.^34–36^ Orthotopic breast cancer represents a relatively superficial tumor type, which is advantageous for direct intratumoral injection as a form of local therapy.^37–39^ Also, it falls within the adequate penetration depth of 730 nm irradiation.^40–42^ More importantly, numerous clinical trials investigating intratumoral drug administration in breast cancer are currently ongoing.^43–45^ Therefore, we established an orthotopic MDA-MB-231 breast tumor mouse model to evaluate the therapeutic effects of the intratumoral Ag_7_ NCs. Briefly, luciferase (Luc)-labeled MDA-MB-231 cells were injected into the left fourth mammary pad of female NOD-SCID mice. The treatments were initiated approximately 1 month later, when tumor volumes exceeded 100 mm^3^, and IVIS could detect bioluminescent signals. Tumor-bearing mice were randomly divided into four groups: intratumoral injection of sterile PBS with/without 730 nm irradiation, or intratumoral injection of 300 ppm Ag_7_ NCs with/without 730 nm irradiation (Figure 4a). Each group received a single treatment on Day 0, followed 24 h later by irradiation with a 730 nm NIR laser (71 mW cm^–2^) for 20 min. Body weight, tumor volume, and bioluminescence signals were continuously monitored.

**Figure 4:**
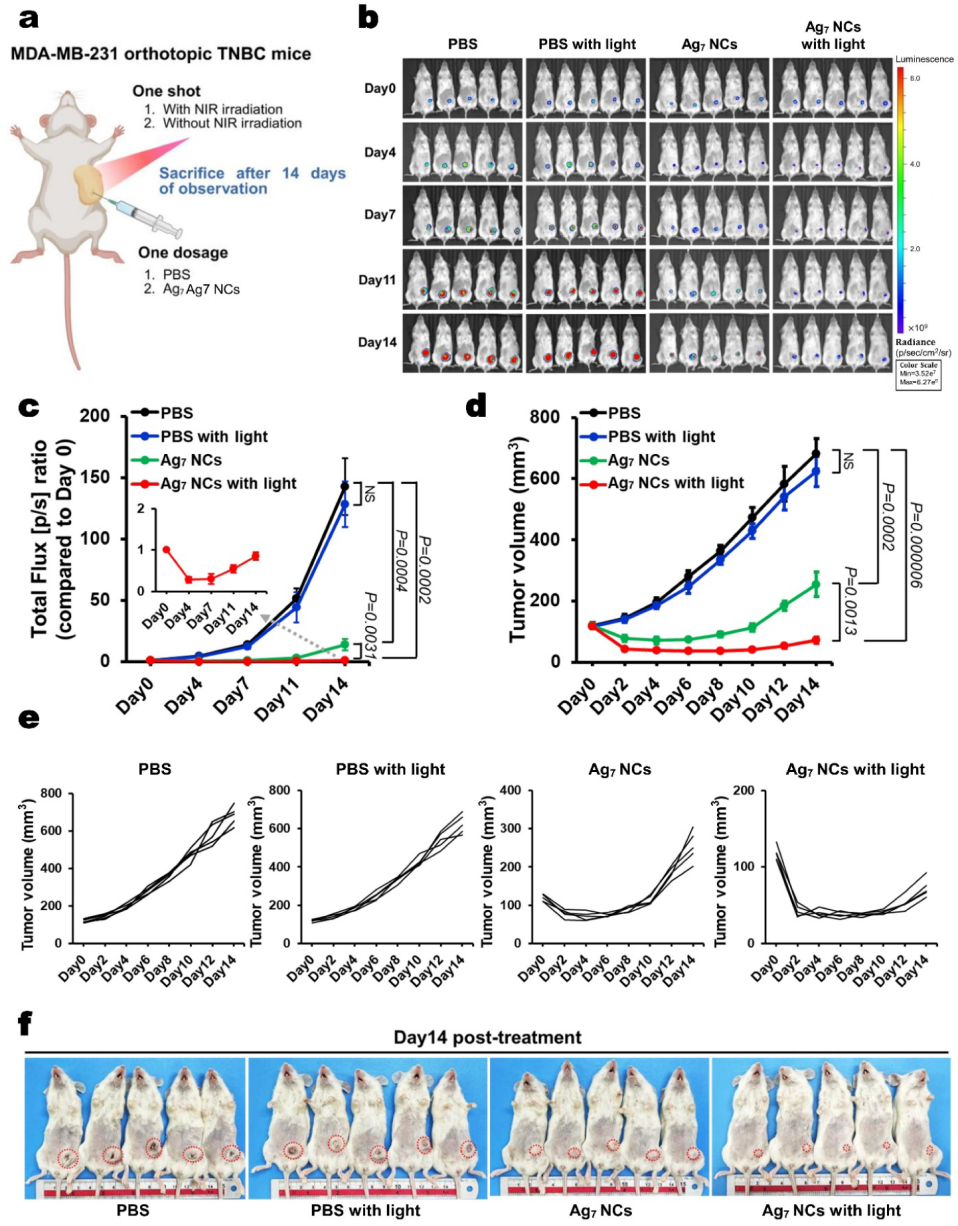
*In vivo* antitumor efficacy of Ag_7_ NCs in orthotopic MDA-MB-231 xenograft mice. (a) Schematic illustration of the treatment procedure created with BioRender.com. MDA-MB-231 cells labeled with luciferase were injected into the left fourth mammary fat pad of female BALB/c mice. Tumor-bearing mice then received an intratumoral injection of either sterile PBS or 300 ppm Ag_7_ NCs, followed by irradiation with a 730 nm NIR laser (71 mW cm^–2^) for 20 min, at 24 h postinjection. All treatments were administered as a single dose. (b) *In vivo* bioluminescence of MDA-MB-231 tumors in each group was monitored by the IVIS system (*n* = 5). (c) Bioluminescence values were measured as the total flux ratio on post-treatment Days 0, 4, 7, 11, and 14 relative to Day 0 in each group (*n* = 5). Data is presented as mean ± SD, with p-values calculated using one-way ANOVA followed by Tukey’s multiple comparisons test. (d), (e) Tumor volumes in each group were measured every 2 days using a caliper and calculated according to the formula: volume = (width^2^ × length)/2 (*n* = 5). Data is presented as mean ± SD, with p-values calculated using one-way ANOVA followed by Tukey’s multiple comparisons test. (f) Tumor morphology after 14 days post-treatment in each group (*n* = 5). The red dashed circles indicate the tumor regions.

The results showed that Ag_7_ NCs with light induced significant tumor regression beginning on Day 2 post-treatment, with visibly reduced tumor size compared to PBS and PBS with light groups (Figure 3b–e, Figure S12a). Notably, the Ag_7_ NCs in the light group exhibited greater tumor suppression than Ag_7_ alone. By Day 7, tumors in the Ag_7_ NC group began to regrow rapidly, whereas tumors in the Ag_7_ NCs with light group remained suppressed until Day 14, showing only a slight increase but still below Day 0 levels. No significant changes in body weight were observed in any group, except for minor decreases in PBS and PBS with light groups due to rapid tumor growth. However, none reached humane end point criteria (Figure S12b). On Day 14 post-treatment, ex vivo analysis confirmed that the Ag_7_ NCs with the light group had the lowest bioluminescence activity, the smallest visible tumor size, and the lightest tumor weight (Figure 5a–b). Importantly, bioluminescence signals from common metastatic organs (lungs and liver) were readily detectable in PBS and PBS with light groups but were reduced in Ag_7_ NCs and markedly suppressed in the Ag_7_ NCs with light group (Figure 5c–d), indicating not only inhibition of primary tumor growth but also suppression of metastasis.

**Figure 5:**
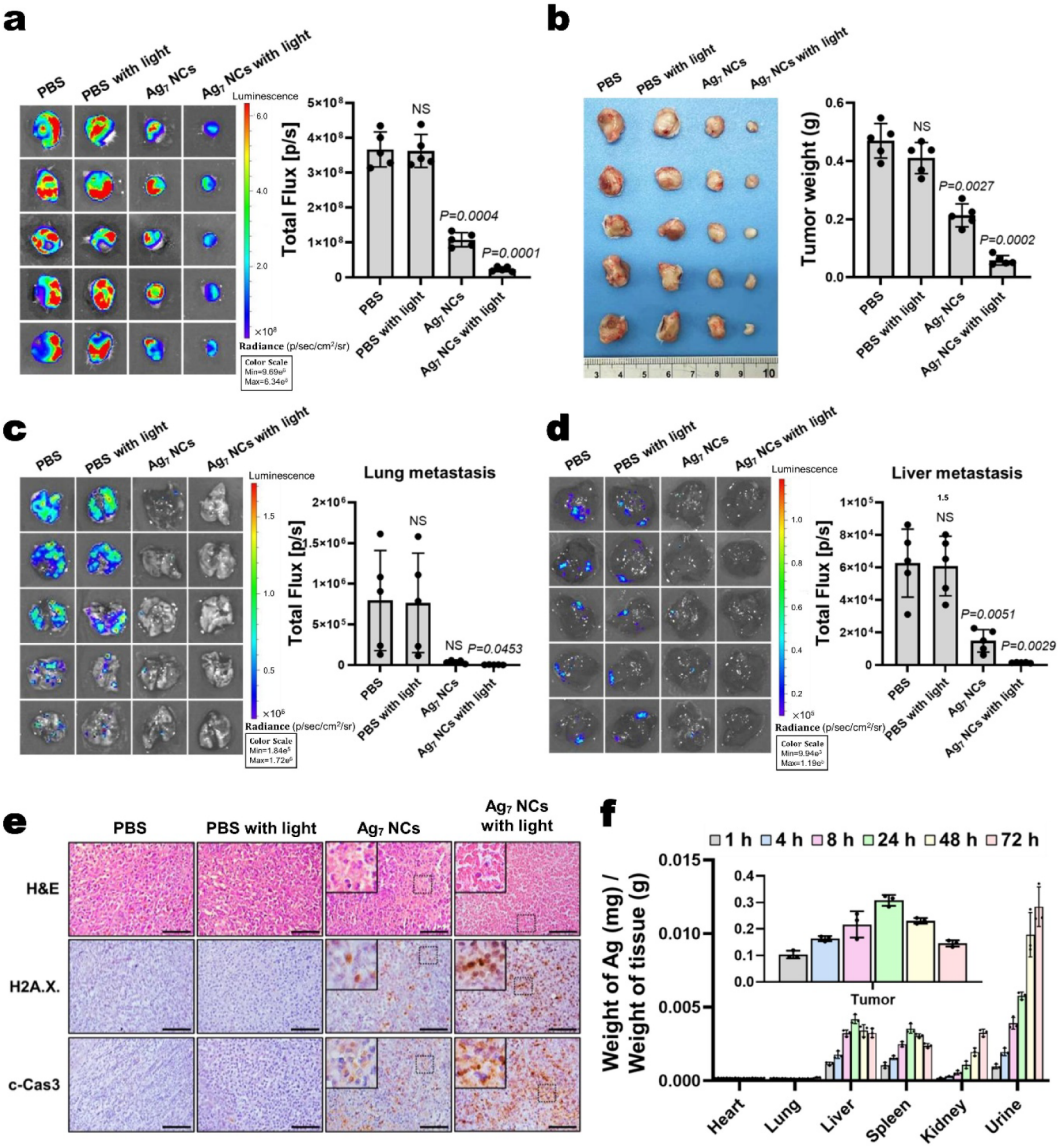
Ag_7_ NCs nanotherapy induces cancer cell death, suppressing tumor growth and metastasis. (a) Ex vivo bioluminescence monitored by the IVIS system and (b) gross appearance of MDA-MB-231 tumors from each group on Day 14 post-treatment (*n* = 5). Data are presented as mean ± SD, with p-values calculated using one-way ANOVA followed by Tukey’s multiple comparisons test. (c) Ex vivo bioluminescence of isolated lungs and (d) livers from each group on Day 14 post-treatment (*n* = 5). Data are presented as mean ± SD, with p-values calculated using one-way ANOVA followed by Tukey’s multiple comparisons test. (e) Histological morphology and expression of phospho-histone H2A.X and cleaved Caspase-3 (Asp175) assessed by H&E and IHC staining (scale bar, 100 μm). Dashed boxes indicate zoomed-in regions. Experiments were independently repeated at least three times with consistent results, and one representative experiment is shown. (f) Biodistribution of Ag_7_ NCs at different post-treatment time points (1, 4, 8, 24, and 72 h) in tumors, heart, lungs, liver, spleen, kidneys, and urine from orthotopic MDA-MB-231 xenograft mice that received an intratumoral injection of 300 ppm Ag_7_ NCs (*n* = 3).

Next, histological analyses further validated these findings. Tumors from the Ag_7_ NCs with the light group showed the most prominent signs of cancer cell death, including the fewest viable nuclei, as well as strong expression of the ROS-induced DNA damage marker phospho-H2A.X and the apoptosis marker cleaved Caspase-3, accompanied by higher H-scores (Figure 5e, Figure S13). Notably, HIF-1α expression was significantly reduced in the Ag_7_ NCs group, indicating intratumoral O_2_ generation and effective hypoxia alleviation (Figure S14). In contrast, in the Ag_7_ NCs + light group, the regenerated O_2_ was consumed to generate ^1^O_2_ during NIR irradiation, leading to an increased HIF-1α expression compared to the nonirradiated group, reflecting enhanced oxygen consumption under light activation. However, HIF-1α levels in this group still showed a slight reduction compared to the PBS and light-only groups, suggesting a diminished oxygen-replenishing effect under irradiation due to concurrent O_2_ utilization for ROS generation. Importantly, although the oxygen-replenishing capacity is partially offset by light exposure, the generated ^1^O_2_ exhibits markedly enhanced cytotoxicity, thereby contributing to more effective tumor cell killing. In future studies, we aim to develop *in vivo* imaging techniques to track intratumoral oxygen changes.

### *In Vivo* Biocompatibility of Ag_7_ NCs

To further assess the translational potential of this therapeutic strategy, we evaluated the biodistribution of Ag_7_ NCs in tumor-bearing mice. Following intratumoral injection of 300 ppm Ag_7_ NCs into orthotopic MDA-MB-231 breast cancer mice, tumor tissues, major organs, and urine samples were collected at different time points. Inductively Coupled Plasma (ICP) analysis revealed that the nanoclusters remained stably localized within the tumor, reaching a peak accumulation at 24 h postinjection and gradually declining thereafter (Figure 5f). This may be attributed to the fact that tumor vasculature is often irregular, tortuous, and highly permeable, and the presence of blood vessels surrounding the mammary gland likely facilitates partial leakage into the systemic circulation.^46^ Consequently, the primary clearance route was identified as renal excretion, as evidenced by a time-dependent increase in Ag_7_ NCs concentration in the kidney and urine. In addition, metabolism was observed in the liver and spleen. These findings indicate that Ag_7_ NCs and mainly eliminated via the urinary pathway.

Finally, we assessed the biosafety of Ag_7_ NCs under 730 nm irradiation in healthy mice. Female BALB/c mice received a 300 ppm intramammary injection of Ag_7_ NCs into the left fourth mammary pad, followed by NIR laser irradiation (71 mW cm^–2^, 20 min) after 24 h. No significant body weight changes were observed over 7 days (Figure S15a). Histopathological examination of major organs also showed no evidence of tissue damage (Figure S15b). Blood and urine biochemical analyses revealed no abnormalities compared with PBS controls (Figure S15c–d). These results demonstrate that local treatment with Ag_7_ NCs combined with NIR irradiation exhibits excellent biosafety *in vivo*, supporting its potential for further clinical development as a practical therapeutic approach for breast cancer.

The absence of acute histopathological changes (Figure 5e) associated with organs combined with no Ag species released upon dialysis (<0.015% over 7 days) (Figure S6f), suggests that Ag_7_ NCs minimize the risk of chronic silver accumulation and associated side effects like argyria. Given the rapid renal clearance (Figure 5f) and high chemical stability (Figure S6) demonstrated, we believe these clusters follow a “fast-in, fast-out” safety profile that differentiates them from larger persistent silver nanoparticles. While the absence of dialyzable silver release, rapid renal clearance and biosafety profile observed in the study suggests a favorable safety margin, further sub chronic toxicity studies and long-term pharmacokinetics will be conducted in future work to fully evaluate the clinical potential and long-term biocompatibility of these nanoclusters.

This Ag_7_ NC system differs from conventional engineered nanomaterials that regulate tumor progression through immune reprogramming, metabolic disruption, or amplification of DNA damage.^47–49^ Compared with the recently reported systems,^47–49^ which rely on complex heterostructure engineering, including polarization-modulated piezocatalysts, ECM-remodeling sonopiezoelectric platforms, and phase-engineered photothermoelectric nanozymes, the Ag_7_ NCs offer a compositionally minimal yet functionally integrated cluster system. This structurally simpler Ag_7_-MBISA architecture enables chemically and phototriggered ROS regulation without requiring bulk semiconductor phase engineering, multicomponent heterointerfaces, or heat-assisted enzyme-mimetic amplification. It also differs from previously reported silver nanocluster-based cancer therapies,^50–53^ which typically rely on single-mode mechanisms, such as ROS generation or imaging, and often lack structural robustness and controllable dual-functional behavior. In contrast, our work highlights a distinct advantage over conventional Ag nanocluster systems by integrating (i) dual-mode and stimulus-dependent ROS generation, and (ii) enhanced stability and *in vivo* applicability, thereby providing a new paradigm for precision nanomedicine in cancer therapy.

## Conclusions

In summary, ultrasmall Ag_7_ NCs provide a dual-mode cancer nanotherapy by exhibiting complementary ROS activity under light and dark conditions. Their ability to generate singlet oxygen under NIR irradiation and hydroxyl radicals in darkness enables stimulus-dependent activation of oxidative stress, achieving selective cytotoxicity toward cancer cells while sparing normal tissues. Combined with the observed renal clearance and absence of acute tissue toxicity, the absence of silver release profile supports the favorable short-term safety of Ag_7_ NCs, although extended subchronic toxicity studies will still be required for future translational evaluation. We are currently working toward advancing to studies in large animal models. The *in vivo* studies confirm significant tumor suppression and metastasis inhibition without systemic toxicity. These findings establish Ag_7_ NCs as a robust and versatile nanoplatform that advances precision cancer therapy through a “light-and-shadow” ROS modulation strategy.

## Methods

### Materials

All chemicals were used as received without further purification. Methanol (99.8%), ethanol (99.5%), 1-butanol (99%), deuterium oxide (D_2_O, 99.8%), silver nitrate (AgNO_3_, 99.8%), *N,N*-dimethylformamide (DMF, 99.5%), sodium acetate (98.5%), hydrogen peroxide (H_2_O_2_, 30.0 ∼ 35.5% (w/w) in H_2_O), hydrochloric acid (1 mol·L^–1^, 6 mol·L^–1^), glycerol (99.5%), *N*,*N*,*N*,N-tetramethylethylenediamine (TEMED, 99.0%), acrylamide (99%), N,N′-methylenebis­(acrylamide) (97.0%), and ammonium peroxodisulfate (APS, 99%) were purchased from Wako Pure Chemical Industries Ltd., Japan. 2-mercapto-5-benzimidazolesulfonic acid sodium salt (MBISA, >98.0%), *3*,*3*′,*5*,*5*′-tetramethylbenzidine (TMB, >98.0%), acetic acid (AcOH, >99.5%), 2,2,6,6-tetramethyl-4-piperidone (TEMP, >98.0%), glycine (>99.0%), indocyanine green (ICG), sodium 4,5-dihydroxybenzene-1,3-disulfonate (>98.0%) and tris­(hydroxymethyl)­aminomethane (Tris, >99.0%) were purchased from Tokyo Chemical Industry (TCI), Japan. Sodium borohydride (NaBH_4_, 99%), 9,10-anthracenediyl-bis­(methylene)­dimalonic acid (ABDA, ≥90.0%), and 5,5-dimethyl-1-pyrroline *N*-oxide (DMPO, ≥98.0%) were obtained from Sigma-Aldrich, USA. Pure water was obtained from a distilled water production apparatus (water distillation apparatus, Aquarius RFD250NB, ADVANTEC). High-glucose Dulbecco’s Modified Eagle’s Medium (DMEM) and Fetal Bovine Serum (FBS) were obtained from Gibco. Phosphate-buffered saline (PBS) and antibiotic penicillin–streptomycin were obtained from Caisson. Tris­(4,7-diphenyl-1,10-phenanthroline)­ruthenium­(II) dichloride complex ([Ru­(ddp)_3_]­Cl_2_, C_72_H_48_Cl_2_N_6_Ru) were purchased from Alfa Aesar. Singlet Oxygen Sensor Green (SOSG), Hoechst 33342, calcein AM, and EthD-III were purchased from Invitrogen. Aminophenyl fluorescein (APF) solution (5 mM in DMF) was purchased from Sigma-Aldrich. Annexin V-FITC Apoptosis Detection Kit were obtained from Sigma-Aldrich.

### Preparation of Ag_7_ NCs

Ag_7_ NCs were synthesized via a previously reported one-pot chemical reduction method with slight modifications.^22^ Briefly, an AgNO_3_ aqueous solution (20.38 mg in 5 mL) is added to water (100 mL), and a MBISA aqueous solution (69.19 mg in 2.4 mL) is added with stirring at 650 r·min^–1^. Thereafter, a NaBH_4_ aqueous solution (13.62 mg in 5 mL of cold water) is rapidly added with stirring at 650 rpm. The solution color immediately changes from yellow to brown to brownish-black. The reaction is further stirred for 5 h. The crude product was divided into five equal portions, and a mixture of *n*-BuOH (16 mL) and MeOH (4 mL) was added to each portion. The upper layer was removed after centrifugation (6000 rpm, 5 min). The upper layer was then removed by centrifugation in the same manner using *n*-BuOH (12 mL). This purification with *n*-BuOH was repeated 6 times, and the resulting precipitate was dried under reduced pressure.

### Characterization of Ag_7_ NCs

The optical and structural characterization of the synthesized Ag_7_ NCs was carried out using various analytical techniques. Ultraviolet–visible (UV–vis) absorption spectra were recorded using a UV–vis–NIR spectrophotometer (V-670, JASCO, Japan). Surface ligands of Ag_7_ were identified by Fourier-transform infrared (FT-IR) spectroscopy (FTIR-4200, JASCO Corporation, Japan) using an attenuated total reflection (ATR) accessory (ATR PRO ONE, JASCO Corporation, Japan). Electrospray ionization mass spectrometry (ESI-MS) was used to confirm the molecular composition of Ag_7_ NCs, while polyacrylamide gel electrophoresis (PAGE) confirmed the monodispersity of the product. The resolving capability of the PAGE conditions used in this study has been verified in our previous work,^53^ where Ag NCs with different sizes or compositions could be separated into multiple electrophoretic bands. The hydrodynamic diameter and zeta potential of Ag_7_ NCs were measured at room temperature using a Nano-ZS (Malvern Instruments, Malvern, United Kingdom). To observe the morphology, transmission electron microscopy (TEM) images were acquired using a Hitachi HT7700 operating at 80 kV.

### ^1^O_2_ Generation via Ag_7_ NCs

^1^O_2_ generation was evaluated using ABDA (0.16 mM) as a chemical probe. Solutions of ABDA and Ag_7_ NCs were prepared in either D_2_O or H_2_O and irradiated with 730 nm light at varying intensities. The time-dependent decrease in ABDA absorbance at 379 nm was recorded under different NC concentrations to quantify and compare ^1^O_2_ production efficiencies under each condition. The generation of ^1^O_2_ was also examined by electron spin resonance (ESR) spectroscopy (ESR5000; Magnettech, BRUKER AXS GmbH, Karlsruhe, Germany), using TEMP as spin-trapping agent.

### •OH Generation via Ag_7_ NCs

•OH generation was corroborated by a colorimetric assay using TMB as a chromogenic probe. The reaction between TMB and •OH resulted in a characteristic blue coloration, confirming •OH production even in the dark in the presence of 100 mM H_2_O_2_ and Ag_7_ NCs. The generation of •OH was also examined by ESR spectroscopy, using DEMPO as a spin-trapping agent.

### O_2_ Generation via Ag_7_ NCs

Dissolved O_2_ measurements using a pen-type dissolved oxygen meter (DOF73020, AS ONE Corporation, Japan) demonstrate catalytic O_2_ generation from 10 mM H_2_O_2_ mediated by Ag_7_ NCs. The O_2_ generation was also evaluated using the oxygen-sensitive probe [Ru­(dpp)_3_]­Cl_2_. Ag_7_ NCs (10 ppm) were dispersed in PBS, (pH 6.4) under brief sonication. All the solution used in the reaction were N_2_-purged for 5 h and handled under dark condition. The oxygen-sensitive probe [Ru­(dpp)_3_]­Cl_2_ was added from a 1 mM DMSO stock to 10 μM final concentration. The total volume of reactants was maintained at 1 mL, comprising of PBS, probe, NCs, and were initiated by H_2_O_2_ (10 μM). The tubes were capped (1.5 mL, black), wrapped in foil and allowed to react for 10 min prior to the photoluminescence measurement (λ_ex_ = 450 nm and λ_em_ = 615 nm).

### Evaluation of Colloidal Stability for NCs

The colloidal stability of Ag_7_ was determined under different physiological conditions. For this, Ag_7_ NCs were diluted to 100 ppm in (i) ultrapure H_2_O, (ii) PBS pH 7.0, (iii) PBS pH 6.0, and (iv) DMEM + 10% FBS. For each group, 1 mL aliquots were prepared in Eppendorf tubes, gently mixed with a pipette, and incubated in the dark at 37 °C with 5% CO_2_ in an incubator. Samples were inspected at 0, 1, 3, and 5 days for visible precipitation, color change, or flocculation. Photographs were taken against a white background using identical exposure settings. Hydrodynamic diameters of the Ag_7_ NCs were analyzed at Day 0, 1, 3, and 5 by dynamic light scattering (DLS) after brief ultrasonication to ensure homogeneity; each condition was measured in triplicate. At Day 0 and Day 5, 10 μL of the dispersion was drop-cast onto carbon-coated Cu grids. High-resolution TEM (HRTEM) and selected-area electron diffraction (SAED) were recorded on a field emission JEOL-2100 operated at 200 kV. The Fourier transform (FFT) analysis was performed using Gatan Digital Micrograph.

### Quantification of Time-Dependent Silver Ion Release

The dissolution kinetics and subsequent release of dialyzable silver species from MBISA-functionalized Ag_7_ NCs were evaluated using a membrane diffusion model. To ensure accurate quantification of ionic silver and prevent the leakage of intact nanoclusters, a Biotech grade Cellulose Ester (CE) membrane (MWCO 500–1000 Da, Spectra/Por) was used for dialysis. The membrane was prerinsed extensively in ultrapure water to remove the 0.05% sodium azide preservative before being equilibrated with 10 mM HEPES. HEPES is a noncoordinating, zwitterionic buffer used in cell culture that maintains a stable physiological pH without interfering with the ionic silver species. Consequently, HEPES provides a “chemically transparent” environment that allows for the accurate quantification of the intrinsic stability of the Ag_7_ NCs and the measurement of its ion-release kinetics. Briefly, 5 mL of the MBISA-functionalized Ag_7_ NCs dispersion (200 ppm) in 10 mM HEPES (pH 7.0) was loaded into the prehydrated dialysis membrane. The assembly was immersed in a reservoir containing 200 mL of the identical HEPES buffer. The system was maintained under mild magnetic stirring at room temperature and strictly protected from light to prevent photoinduced oxidation or reduction of the silver species. At predetermined intervals (1, 3, and 7 days), 2 mL aliquots were withdrawn from the external dialysate and replaced with an equal volume of fresh HEPES buffer to maintain volume. Each aliquot was digested with 2 mL of concentrated nitric acid. The total silver concentration in the dialysate was quantified via Inductively Coupled Plasma Mass Spectrometry (ICP-MS). All experiments were performed in triplicate. Since ICP-MS detects total Ag after digestion, the measured signal is best described as total dialyzable silver rather than free Ag^+^ alone.

### Cell Culture

Human breast epithelial cell line (MCF-10A) and breast cancer cell line (MDA-MB-231) were cultured in high-glucose DMEM (supplemented with 10% FBS and 1% penicillin–streptomycin). All cells were maintained at 37 °C in a humidified atmosphere with 5% CO_2_ in an incubator.

### Time-Dependent Cell-Associated Ag

A cell density of 1 × 10^5^ cells/well was seeded in 6-well plates with 1 mL medium/well and cultured overnight to ∼80% confluence. The Ag_7_ NCs were dispersed into prewarmed high-glucose DMEM to the target concentration of 100 ppm. The medium in the well was replaced with 1 mL of Ag_7_ NCs containing medium per well and incubated for the specified times at 37 °C. After removing residual Ag_7_ NCs from the trypsinized cells by washing them with PBS thrice, the cells were digested in aqua regia for 24 h, followed by filtration. Then, the Ag_7_ NCs ingested by cells were quantified by inductively coupled plasma mass spectrometry (ICP-MS) using an Ag standard.

### Cytotoxicity Assay

Cell viability was assessed by the 3-(4,5-dimethylthiazol-2-yl)-2,5-diphenyltetrazolium bromide (MTT) assay in normal breast epithelial MCF-10A cells and breast cancer MDA-MB-231 cells. Briefly, MCF-10A and MDA-MB-231 cells were seeded into 96-well plates at a density of 1 × 10^4^ and 5 × 10^3^ cells per well, respectively and incubated overnight at 37 °C with 5% CO_2_. Ag_7_ NCs were added at final concentrations of 0, 100, 200, 300, 400, and 500 ppm in DMEM medium, and cells were incubated for another 24 h. For light-activated studies, after the Ag_7_ NCs are being incubated for 24 h, the MDA-MB-231 cells were washed thrice with PBS and then irradiated with 730 nm NIR light (71 mW cm^–2^) for 10 or 20 min. Following treatment, the medium was replaced with MTT solution and incubated for 4 h at 37 °C. The resulting formazan crystals were dissolved in 200 μL DMSO with mild shaking and then centrifuged. The supernatant was transferred to a new 96-well plate, and absorbance was measured at 570 nm in an ELISA reader.

### Intracellular O_2_ Detection

O_2_ generation in MDA-MB-231 cells was evaluated using oxygen-sensitive probe [Ru­(dpp)_3_]­Cl_2_ (ruthenium­(II) tris­(4,7-diphenyl-1,10-phenanthroline) dichloride). Cells were seeded in 24-well plates at a density of 2.5 × 10^4^ cells per well and allowed to adhere overnight. Ag_7_ NCs (300 ppm) were added and incubated for 24 h at 37 °C with 5% CO_2_. The cells were washed thrice with PBS, subsequently incubated with [Ru­(dpp)_3_]­Cl_2_ (10 μM) for 30 min at 37 °C in the dark. Excess probe was removed by washing twice with PBS, and nuclei were counterstained with Hoechst 33342 (2 μg mL^–1^, 10 min). After washing with PBS twice, the cells were imaged using a 10× lens in a Nikon confocal fluorescence microscope (Ex/Em: 450/615 nm).

### Intracellular ROS Detection

ROS generation in MDA-MB-231 cells was evaluated using aminophenyl fluorescein (APF; specific for •OH) and Singlet Oxygen Sensor Green (SOSG; specific for ^1^O_2_). Cells were seeded in 24-well plates at a density of 2.5 × 10^4^ cells per well and allowed to adhere overnight. Ag_7_ NCs were added and incubated for 24 h at 37 °C with 5% CO_2_. For light-activated studies, cells were washed thrice with PBS, subsequently irradiated with a 730 nm NIR laser (71 mW cm^–2^) for 10 or 20 min, while dark controls were kept under identical conditions without irradiation. After treatment, cells were incubated with APF (10 μM) or SOSG (5 μM) for 30 min at 37 °C in the dark. Excess probe was removed by washing twice with PBS, and nuclei were counterstained with Hoechst 33342 (2 μg mL^–1^, 10 min). After washing with PBS twice, the cells were imaged using a 10× lens in a Nikon confocal fluorescence microscope. Fluorescence intensity of APF (Ex/Em: 490/515 nm) and SOSG (Ex/Em: 504/525 nm) was quantified.

### Flow Cytometry

Apoptosis was quantified in MDA-MB-231 breast cancer cells using Annexin V-FITC/propidium iodide (PI) dual staining. Cells were seeded in 24-well plates at a density of 2.5 × 10^4^ cells per well and allowed to adhere overnight. Ag_7_ NCs (300 ppm) were added and incubated for 24 h at 37 °C. For light-activated conditions, the cells were washed with PBS twice before being irradiated with 730 nm NIR light (71 mW cm^–2^) for 20 min. At the same time, dark controls were kept under identical conditions without irradiation. Following treatment, cells were harvested by trypsinization, washed twice with cold PBS, and resuspended in 1× Annexin V binding buffer. Aliquots of 100 μL cell suspension were incubated with 2 μL Annexin V-FITC and 1 μL PI for 15 min at room temperature in the dark, then diluted with 400 μL binding buffer before analysis. Flow cytometry was performed with 10,000 events collected per sample. Compensation settings were adjusted using single-stained controls. Data were analyzed, and populations were classified as viable (Annexin V^–^/PI^–^), early apoptotic (Annexin V^+^/PI^–^), late apoptotic/secondary necrotic (Annexin V^+^/PI^+^), or necrotic (Annexin V^–^/PI^+^). Thapsigargin-treated cells were included as a positive control for apoptosis induction.

### Live and Dead Cell Assay

The cytocompatibility of Ag_7_ NCs was evaluated in normal breast epithelial MCF-10A cells using Calcein AM/Hoechst 33342/EthD-III staining. Cells were seeded in 24-well plates at a density of 5 × 10^4^ cells per well and cultured overnight. Cells were treated with 300 ppm Ag_7_ NCs for 24 or 48 h at 37 °C. Untreated cells served as controls. After incubation, cells were washed twice with PBS and stained with Calcein AM (2 μL), Hoechst 33342 (1 μL), and EthD-III (2 μL) in 2 mL DMEM for 20 min at 37 °C in the dark. Stained cells were washed with PBS and immediately imaged using a Nikon confocal fluorescence microscope. Calcein AM identified viable cells, EthD-III marked dead cells with compromised membranes, and Hoechst 33342 counterstained cell nuclei. Representative fluorescence images were obtained from three independent experiments, and scale bars were set at 20 μm. The viability of MDA-MB-231 cells after treatment with Ag_7_ NCs was assessed using a Calcein AM/Hoechst 33342/EthD-III staining kit. Cells were seeded onto 24-well plates at a density of 2.5 × 10^4^ cells per well and cultured overnight. Cells were treated with 300 ppm Ag_7_ NCs for 24 h at 37 °C. For light-activated conditions, cells were washed twice with PBS and then irradiated with 730 nm NIR light (71 mW cm^–2^) for 20 min, while dark and light-only groups were maintained as controls. After treatment, cells were incubated with a staining solution containing Calcein AM (2 μL), Hoechst 33342 (1 μL), and EthD-III (2 μL) in 2 mL DMEM for 20 min at 37 °C in the dark. Cells were washed with PBS and imaged using a Nikon confocal fluorescence microscope. Live cells emitted green fluorescence (Calcein AM), dead cells with compromised membranes emitted red fluorescence (EthD-III), and nuclei were stained blue (Hoechst 33342). Representative images were collected from three independent experiments, and scale bars were set at 20 μm. Fluorescence images were quantified using ImageJ. Cells were classified as live (Calcein AM^+^/EthD-III^–^) or dead (EthD-III^+^) based on intensity thresholds determined from untreated controls, and percentages were calculated from at least three random fields per condition across three independent experiments.

### Hemolysis Testing

Fresh sheep red blood cells (RBCs) were precleaned prior to hemolysis testing. Briefly, RBCs were pelleted by centrifugation at 1200 rpm for 3 min at 4 °C, and the supernatant was discarded. The RBC pellet was resuspended in PBS and washed repeatedly (centrifugation/resuspension cycles) until the supernatant became clear, then diluted in PBS to a 2% (v/v) working suspension. For the hemolysis assay, 2% RBCs were incubated with Ag_7_ NCs (10 ppm) for 30 min at room temperature in the dark. PBS and deionized water (DDW) served as the negative (0% hemolysis) and positive (100% hemolysis) controls, respectively. Following incubation, samples were centrifuged to pellet intact RBCs, and 100 μL of the supernatant was transferred to microplate. The absorbance of released hemoglobin in the supernatant was measured at 540 nm (OD_540nm_) using the microplate reader. To correct for nanocluster-related absorbance overlap, OD_540nm_ values were background-subtracted using Ag_7_ dispersed in PBS without RBCs under identical conditions (*Absorbance*_*sample*_ *= Absorbance*_Ag_7_+RBC_ – *Absorbance*_Ag_7_+PBS_). Percent hemolysis was calculated as *%Hemolysis =* (*Absorbance*_*sample*_ – *Absorbance*_(−)*control*_)/(*Absorbance*_(+)*control*_ *– Absorbance*_(−)*control*_) × 100. All measurements were performed in triplicate and are reported as mean ± s.e.m.

### *In Vivo* Therapeutic Evaluation

All animal experiments were conducted in accordance with the Laboratory Animal Welfare Act and the Guide for the Care and Use of Laboratory Animals, with approval from the Institutional Animal Care and Use Committee (IACUC) of National Cheng Kung University (NCKU) (IACUC No. 114282). The NCKU Laboratory Animal Center guidelines strictly follow animal handling and surgical procedures. Experimental mice were housed in groups of three to five per cage under controlled conditions (22–23 °C, 55 ± 10% humidity) with a 13 h/11 h light/dark cycle. For the orthotopic MDA-MB-231 breast cancer xenograft model, female NOD-SCID mice (6–8 weeks) were injected with a total of 1 × 10^7^ MDA-MB-231-Luc cells suspended in 20 μL of PBS/Matrigel (1:1) into the left fourth mammary fat pad. The needle was retained in place for 30 s before withdrawal to minimize leakage. After approximately 1 month, tumor-bearing mice were randomly assigned to receive a single intratumoral injection of 100 μL sterile PBS or 100 μL of 300 ppm Ag_7_ NCs suspended in sterile PBS. After 24 h, the tumor area was irradiated with a 730 nm NIR laser (71 mW cm^–2^) for 20 min. Subsequently, the body weight, tumor volume, and bioluminescence signals of the experimental mice were continuously monitored.

### IVIS Detection

Mice were anesthetized with oxygen and isoflurane and administered an intraperitoneal injection of 100 μL D-luciferin (#122796, Caliper Life Sciences). For *in vivo* imaging, mice were first anesthetized and then imaged using the IVIS Spectrum Imaging System (IVIS; P/N 124262, Caliper Life Sciences) approximately 10 min postinjection, after luciferin distribution and full sedation. Imaging was performed sequentially, and each mouse was allowed to recover before the next group was processed. Although imaging time points varied between groups due to the system capacity, luciferin incubation time and imaging conditions were kept consistent. For ex vivo imaging, tumors and organs were collected after euthanasia, incubated with luciferin for ∼1 min, and imaged individually. Images were analyzed using Living Image software (v4.7.3, Caliper Life Sciences), with identical luminescence thresholds applied across all groups to ensure consistent quantification and enable direct comparisons under a uniform baseline.

### Hematoxylin and Eosin (H&E) Staining

Tumors and normal organ samples (heart, lung, spleen, liver, and kidney) were paraffin-embedded and sectioned at a thickness of 5 μm. Sections were deparaffinized, rehydrated, washed with PBS, and stained with hematoxylin (#GHS316, Merck) for 3 min, followed by eosin (#HT110232, Merck) for 1 min. After dehydration with ethanol (#100983, Merck) and clearing with xylene (#108298, Merck), samples were mounted for evaluation. Stained slides were examined using a BX51 microscope (Olympus), and three representative fields per group were imaged.

### Immunohistochemistry (IHC) Staining

Tumor samples were paraffin-embedded and sectioned at 5 μm thickness. After deparaffinization and rehydration, sections were incubated with primary antibodies against Phospho-Histone H2A.X (#2577, Cell Signaling Technology; 1:400), Cleaved Caspase-3 (#9661, Cell Signaling Technology; 1:400), and HIF-1 alpha (#36169, Cell Signaling Technology; 1:400). Immunostaining was conducted using an ABC peroxidase kit (#32020, Thermo Fisher Scientific) and visualized with a DAB substrate kit (#SK-4100, Vector Laboratories) according to the manufacturer’s instructions. Stained sections were examined using a BX51 microscope (Olympus), and three representative fields per group were imaged. For the quantification, QuPath software (v0.7.0) was used to calculate H-scores for each field of view across the different groups.

### Biodistribution Analysis

Tumor tissues, major organs (heart, lung, spleen, liver, and kidney), and urine samples were collected, washed twice with PBS, and weighed. Tissues were homogenized into powder and digested in aqua regia for 1 week. The silver (Ag) content in the digested samples was quantified using an iCAP 7400 ICP-OES system (Thermo Fisher Scientific).

### Biochemical Analysis

Blood was collected from the hearts of mice into tubes containing heparin sodium (#HY-17567, MedChemExpress) and centrifuged at 3000 rpm for 10 min to obtain serum. Serum samples were analyzed for alanine aminotransferase (ALT), alkaline phosphatase (ALP), aspartate aminotransferase (AST), total bilirubin (T-Bil), blood urea nitrogen (BUN), creatinine (CREA), and uric acid (UA) using an automated biochemical analyzer (FUJI DRI-CHEM 4000i, FUJIFILM). Urine samples were diluted 1:5 and analyzed using a urine analyzer (RT-4010, Arkray) for glucose, protein, bilirubin, pH, blood, ketones, nitrite, leukocytes, creatinine, and albumin.

### Statistical Analysis

All experiments were performed independently at least in triplicate, and results are presented as mean ± SEM or SD. Group differences were evaluated using a one-way ANOVA followed by Tukey’s multiple comparisons test for post hoc comparisons, with *P* < 0.05 considered statistically significant.

## Supplementary Material



## Notes

ESI-MS spectrum of Ag_7_ NCs and the simulated isotope pattern; high-resolution S 2p XPS spectrum of Ag_7_ NCs; morphological and structural characterization of Ag_7_ NCs by HRTEM; determination of the singlet oxygen quantum yield (Φ) of Ag_7_ NCs under 730 nm irradiation using ICG as a reference photosensitizer and ABDA as a ^1^O_2_ trapping probe; time-dependent UV–vis absorption spectra of ABDA under 730 nm irradiation in the presence of Ag_7_ NCs; colloidal stability of Ag_7_ NCs; ABDA-based evaluation of singlet oxygen generation under 730 nm irradiation; X-ray photoelectron spectroscopy (XPS) and Auger spectral profile of Ag_7_ NCs before and after H_2_O_2_ exposure; Ag MNN Auger spectra of Ag_7_ NCs measured before and after treatment with 10 mM H_2_O_2_ under dark conditions; live/dead cell staining of MCF-10A normal cells treated with Ag_7_ NCs for 24 and 48 h; hemolysis assessment of Ag_7_ NCs*; in vivo* evaluation of Ag_7_ NC treatment—Tumor morphology after 2 days post-treatment and body weight data; H-score analysis of H2A.X. and c-Cas3; validation of intratumoral hypoxia relief and self-oxygenated PDT; *in vivo* biosafety of Ag_7_ NCs—Body weight, histological morphology of the heart, lung, liver, spleen, and kidney, as well as serum and urine biochemical parameters after 7 days post-treatment (PDF)
