# Supplementary material for: Ultrasmall Ligand-Protected Ag7 Nanoclusters Enable Dual-Mode Reactive Oxygen Species Generation under Dark and Near-Infrared Irradiation
Source: ACS Nano. 2026 May 28;20(22):16118–34. doi: 10.1021/acsnano.6c01532 (PMC13255526; doi:10.1021/acsnano.6c01532)
Supplement: Supplementary file 1 [file nn6c01532_si_001.pdf]

Supporting Information

Ultrasmall Ligand-Protected Ag_7 Nanoclusters Enable Dual-Mode Reactive Oxygen Species Generation under Dark and Near-Infrared Irradiation

Divinah Manoharan,[†] Kana Yamamoto,[‡] Li-Chan Chang,[ƻ] Chouma Kurihashi,[§] Issey Osaka,[§] Yin-Fen Liu,[ƻ] Siou-Wei Liang,[†] Hideya Kawasaki,[‡] Wen-Pin Su,[ƻ‖±]*, Chen-Sheng Yeh[†‖]**

[†]Department of Chemistry, National Cheng Kung University, Tainan 701, Taiwan.

[‡]Department of Chemistry and Materials Engineering, Kansai University, 3-3-35 Yamate-cho, Suita, Osaka 564-8680, Japan.

[ƻ]Institute of Clinical Medicine, College of Medicine, National Cheng Kung University, Tainan 704, Taiwan

[§]Department of Pharmaceutical Engineering, Faculty of Engineering, Toyama Prefectural University, Imizu, Toyama 939-0398, Japan

[‖]Center of Applied Nanomedicine, National Cheng Kung University, Tainan 701, Taiwan

[±]Departments of Oncology and Internal Medicine, National Cheng Kung University Hospital, College of Medicine, National Cheng Kung University, Tainan 704, Taiwan.

Figure S1

(a)

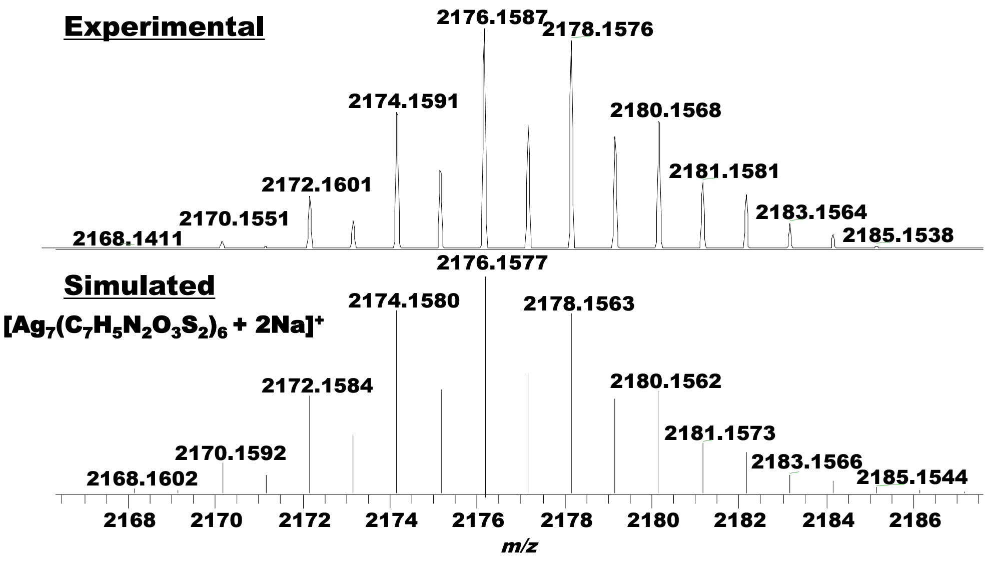

Experimental
2176.1587
2178.1576
2174.1591
2180.1568
2172.1601
2181.1581
2170.1551
2183.1564
2168.1411
2185.1538
Simulated
[Ag7(C7H5N2O3S2)6 + 2Na]+
2176.1577
2174.1580
2178.1563
2172.1584
2180.1562
2181.1573
2170.1592
2183.1566
2168.1602
2185.1544
2168
2170
2172
2174
2176
2178
2180
2182
2184
2186
m/z

(b)

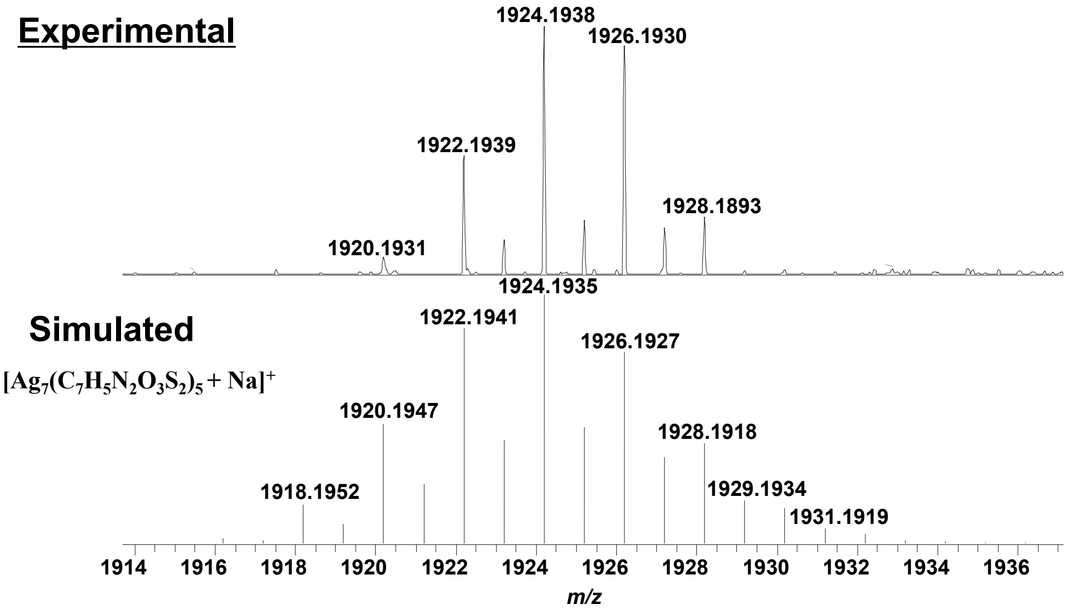

Experimental
1924.1938
1926.1930
1922.1939
1928.1893
1920.1931
Simulated
[Ag7(C7H5N2O3S2)5 + Na]+
1924.1935
1922.1941
1926.1927
1920.1947
1928.1918
1918.1952
1929.1934
1931.1919
1914
1916
1918
1920
1922
1924
1926
1928
1930
1932
1934
1936
m/z

Figure S1. In the ESI–MS spectrum, a dominant peak is observed at *m/z* ≈ 2176, which is consistent with the $Ag_7(MBISA)_6$ cluster composition (S1a). In addition to this dominant peak, several weaker peaks appear in the intermediate mass region (*m/z* ≈ 1500–2000). These peaks can be reasonably assigned to ligand-loss fragments such as $Ag_7(MBISA)_5$ (S1b) generated during the electrospray ionization and desolvation processes. Such ligand-loss is commonly observed in ESI-MS measurements of ligand-protected metal nanoclusters and are generally attributed to partial ligand dissociation in the gas phase rather than to distinct nanocluster species present in solution. Because silver possesses two naturally abundant isotopes (^{107}Ag and ^{109}Ag), Ag-containing clusters typically exhibit a characteristic isotopic pattern with a regular spacing of approximately 2 Da. The peaks observed below *m/z* ≈ 1400 do not show this characteristic isotopic distribution and are therefore unlikely to originate from Ag-containing clusters. Instead, they are more reasonably attributed to ligand-derived fragments or related organic species generated during the ionization process. Attempts in negative-ion ESI–MS did not yield stable cluster signals, likely due to strong solvation effects of the sulfonate groups.

Figure S2

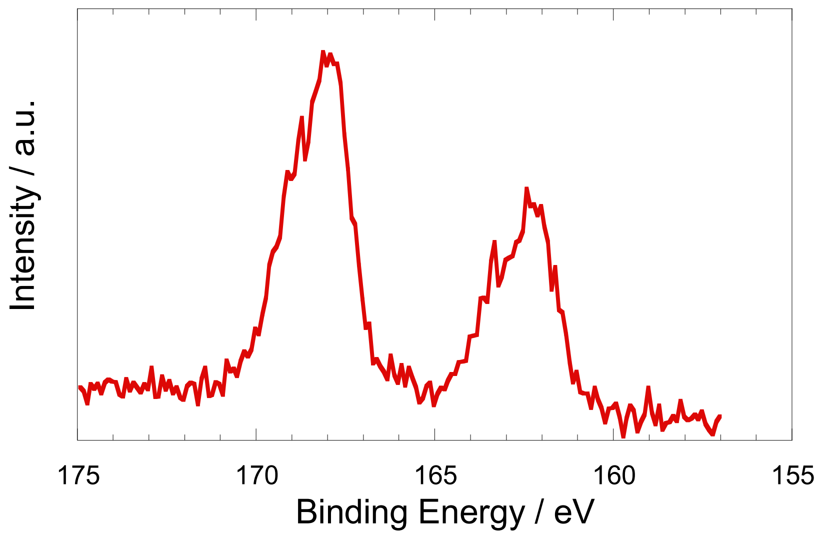

Figure S2. High-resolution S 2p XPS spectrum of Ag_7 NCs. The spectrum was deconvoluted into the Ag–S thiolate bonds (~162.1 eV), and sulfonate groups of MBISA (~167.9 eV).

Figure S3

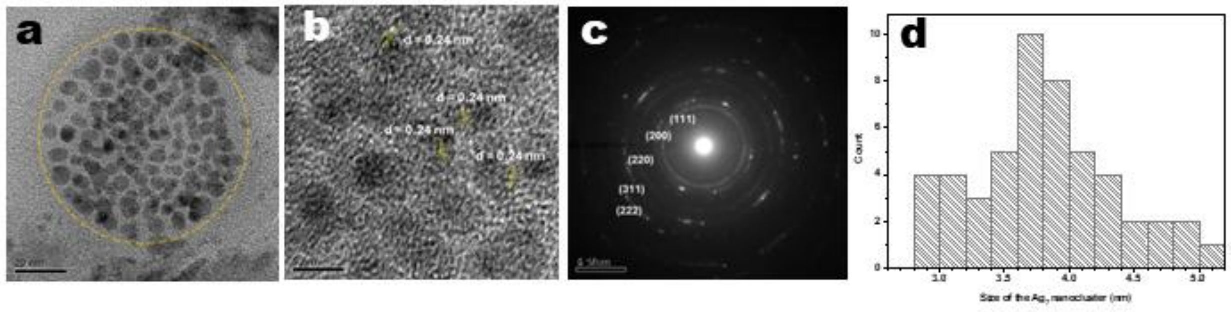

Figure S3. Morphological and structural characterization of Ag_7 NCs. (a), (b) HRTEM image inducing aggregation and coalescence into larger nanoparticles for Ag_7 NCs with uniform and well-dispersed spherical particles. (c) SAED pattern displaying diffraction rings corresponding to the face-centered cubic (fcc) structure of silver. (d) Size distribution histogram (3.8 ± 0.5 nm) derived from multiple HRTEM images using ImageJ software.

Figure S4

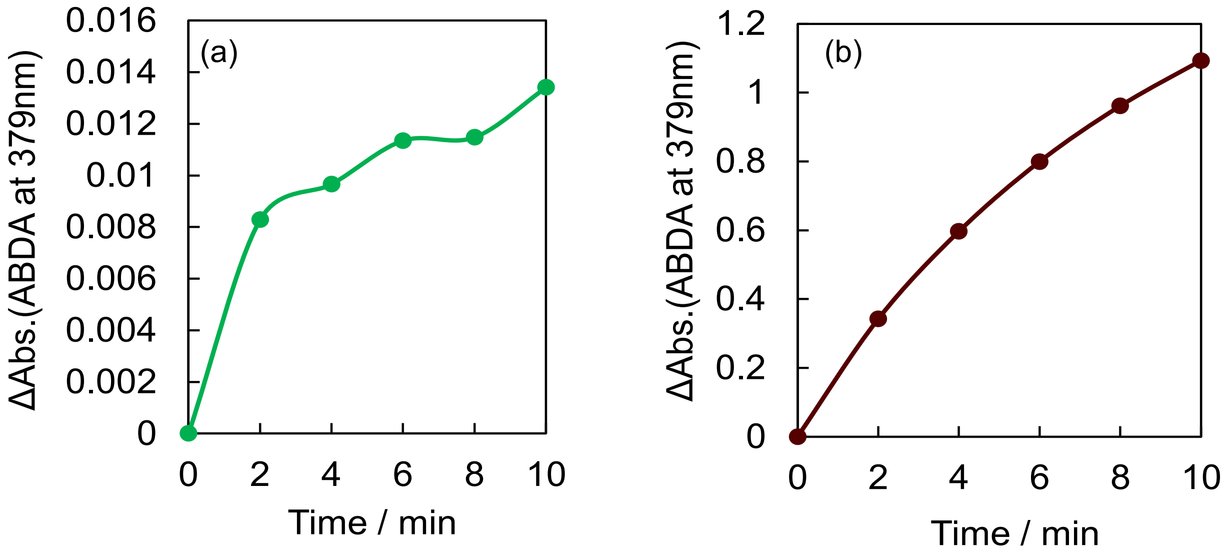

Figure S4. Determination of the singlet oxygen quantum yield (Φ) of Ag_7 NCs under 730 nm irradiation using ICG as a reference photosensitizer and ABDA as a $^1O_2$ trapping probe. Time-dependent changes in the absorbance of ABDA at 379 nm were monitored for (a) ICG and (b) Ag_7 NCs. The *Φ* value of Ag_7 NCs was calculated from the relative ABDA bleaching rates, with ICG used as the reference standard (*Φ*=0.2 %), and was determined to be 8.4%.

Figure S5

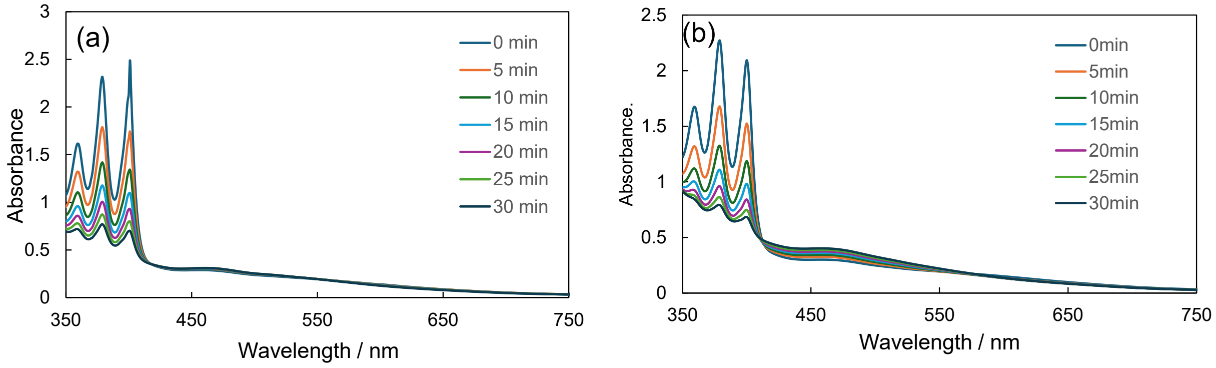

Figure S5 Time-dependent UV–vis absorption spectra of ABDA under 730 nm irradiation in the presence of Ag_7 NCs: (a) without sodium 4,5-dihydroxybenzene-1,3-disulfonate and (b) with sodium 4,5-dihydroxybenzene-1,3-disulfonate (5 mM). The decrease in the characteristic absorption of ABDA at 379 nm indicates $^1O_2$ generation over time.

Figure S6

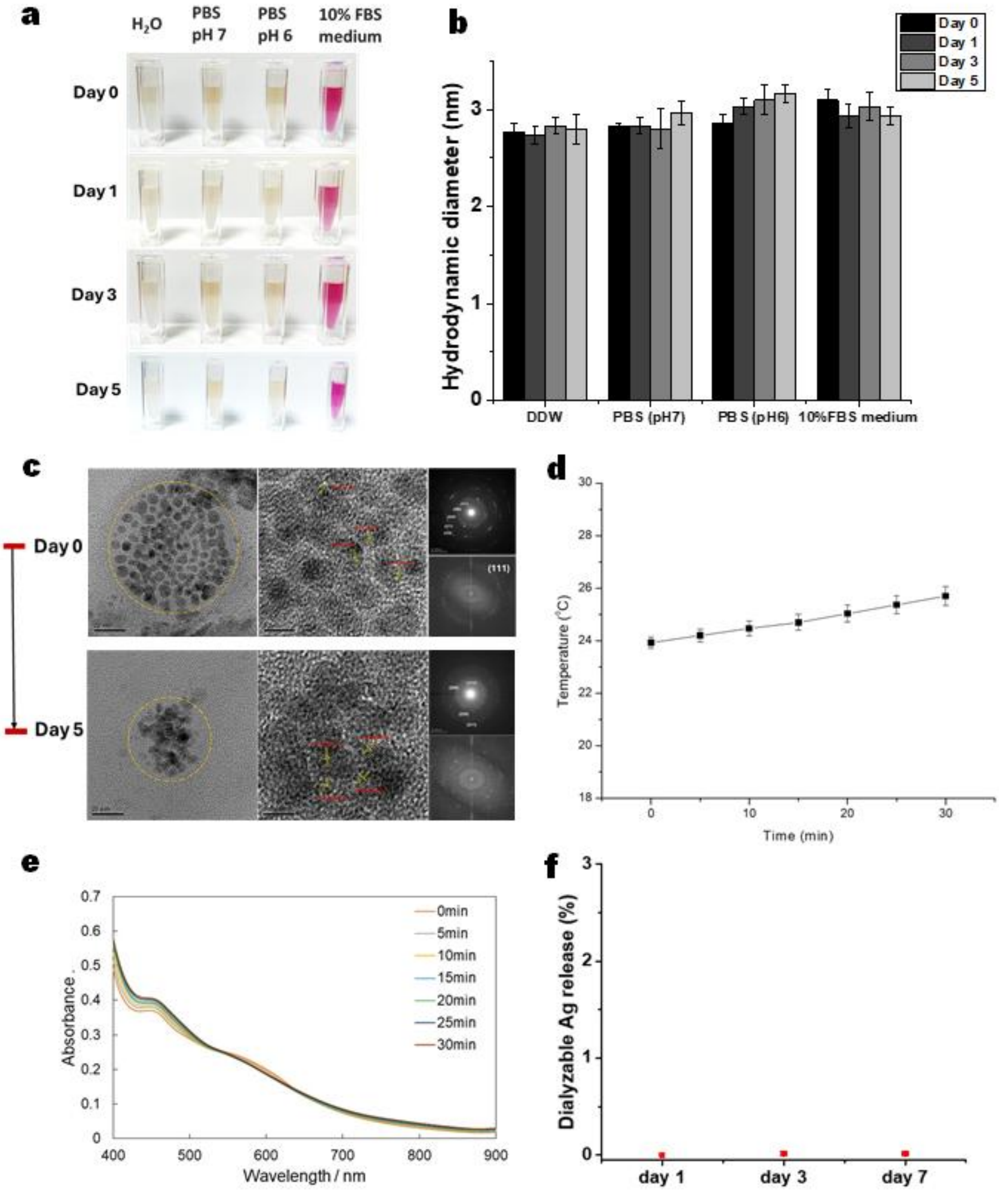

a
H_2O
PBS pH 7
PBS pH 6
10% FBS medium
Day 0
Day 1
Day 3
Day 5
b
Hydrodynamic diameter (nm)
Day 0
Day 1
Day 3
Day 5
DDW
PBS (pH7)
PBS (pH6)
10%FBS medium
c
Day 0
Day 5
(111)
d
Temperature (°C)
Time (min)
e
Absorbance
Wavelength / nm
0min
5min
10min
15min
20min
25min
30min
f
Dialyzable Ag release (%)
day 1
day 3
day 7

Figure S6. Colloidal stability of Ag_7 NCs. (a) Representative photographs of Ag_7 NCs over a course of 5 days dispersed in different physiological medium namely, H_2O, PBS pH 7, PBS pH6 and DMEM medium. (b) Hydrodynamic diameter of Ag_7 NCs measured by dynamic light scattering (DLS) over a course of 5 days dispersed in different physiological medium namely, H_2O, PBS (pH 7), PBS (pH6) and DMEM medium. Data are mean ± s.e.m. (c) HRTEM images taken to observe the corresponding SAED pattern and FFT showing crystal lattice features of Ag_7 NCs at day 0 and 5. (d) Time-dependent temperature increase of 100 ppm Ag_7 NCs dispersed in PBS with 71 mW/cm^2 730 nm red light irradiation (n =3, independent trials). (e) UV–vis spectra during 30 min. NIR irradiation with 71 mW cm^{-2} 730 nm showing stable optical properties of Ag_7 NCs. (f) Time-dependent release of dialyzable silver species from MBISA-functionalized Ag_7 NCs in 10 mM HEPES buffer under dark conditions. The released silver was quantified by ICP-MS after dialysis and acid digestion. Cumulative Ag release from Ag_7 NCs dialyzed against HEPES are expressed as the percentage of total initially loaded silver. Data are presented as mean ± s.e.m. (n = 3).

Figure S7

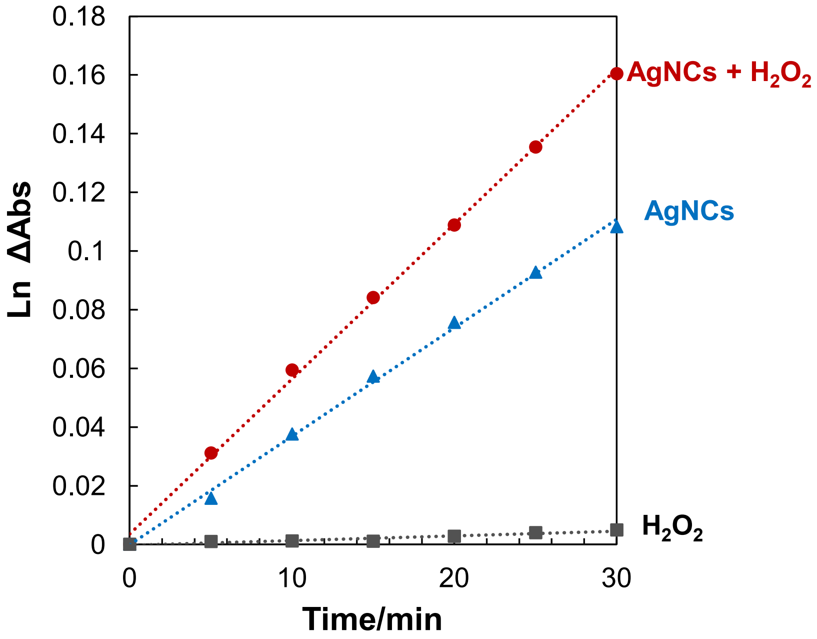

Figure S7. ABDA-based evaluation of singlet oxygen generation under 730 nm irradiation. Time-dependent changes in ln{Abs(0 min.)/Abs(x min.)} at 379 nm for ABDA in the presence of Ag_7 NCs with 10mM H_2O_2, Ag_7NCs alone, 10mM H_2O_2 alone. The enhanced ABDA decay observed for the Ag NCs + H_2O_2 system indicated increased $^1O_2$ generation.

Figure S8

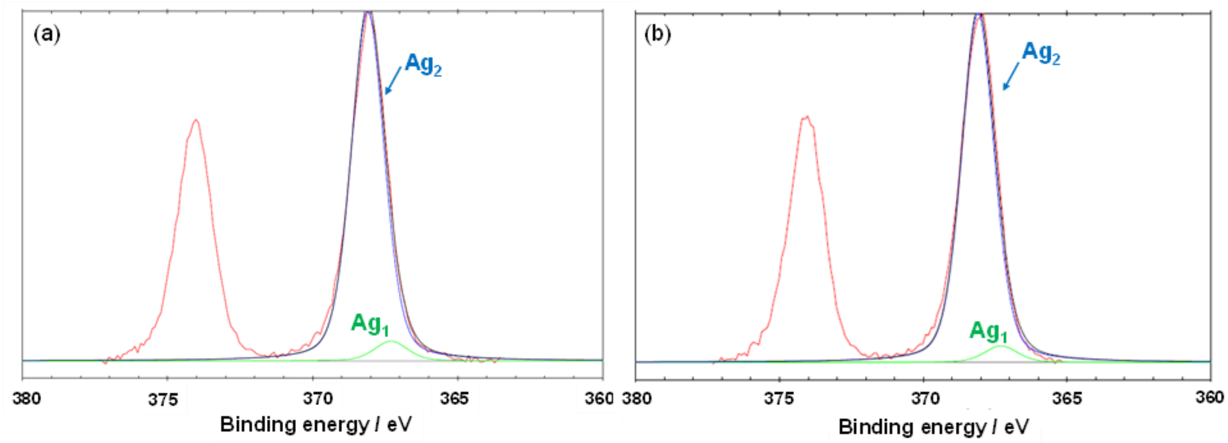

Figure S8. X-ray photoelectron spectroscopy (XPS) of Ag_7 NCs before and after H_2O_2 exposure. Ag 3d spectra of Ag_7 NCs (a) before H_2O_2 treatment and (b) after immersion in 10 mM H_2O_2 for 10 min under dark conditions. In both spectra, the Ag $3d_{5/2}$ peak can be deconvoluted into two components located at 368.1 and 367.3 eV. The relative contributions of the lower- and higher-binding-energy components are 5.3% and 94.7%, respectively, before H_2O_2 exposure and remain essentially unchanged (4.5% and 95.5%) after treatment, indicating that the overall electronic-state distribution of Ag atoms in the nanocluster is largely preserved during H_2O_2 exposure.

Figure S9

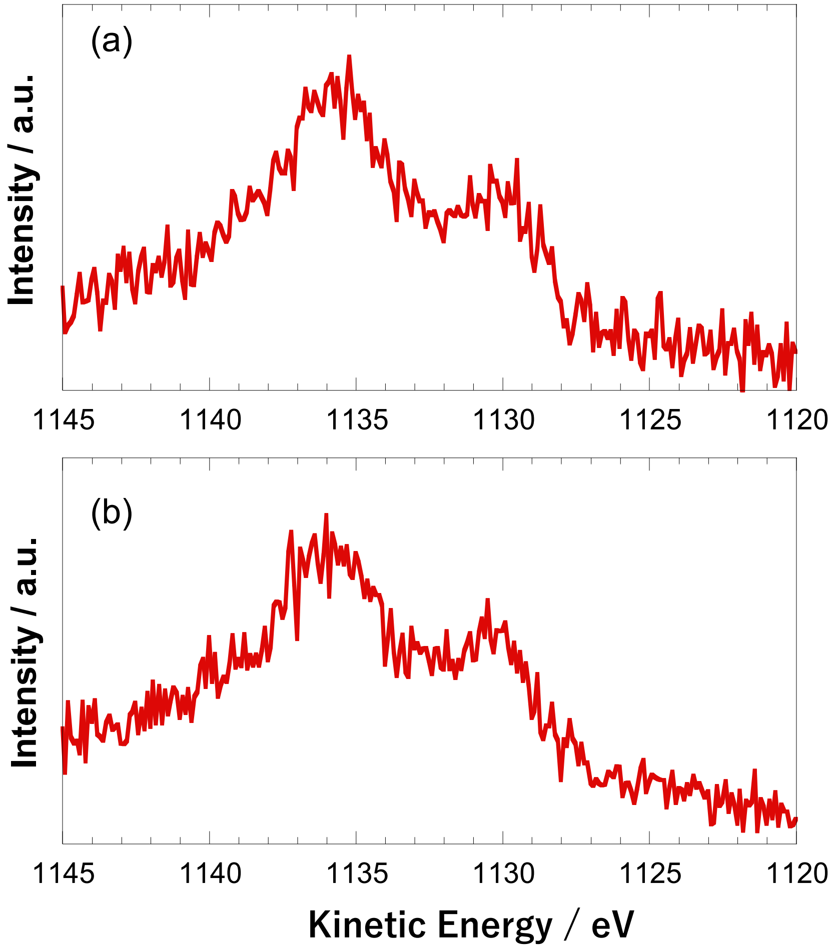

Figure S9. Ag MNN Auger spectra of Ag_7 NCs measured before (top) and after (bottom) treatment with 10 mM H_2O_2 under dark conditions. The spectra exhibit a broad, asymmetric line shape with discernible shoulder features rather than a single symmetric peak, suggesting the presence of non-equivalent Ag environments within the ultrasmall nanocluster structure. Importantly, the overall spectral profile remains essentially unchanged after H_2O_2 exposure, indicating that the electronic structure of the Ag_7 nanoclusters is largely preserved during the reaction.

Figure S10

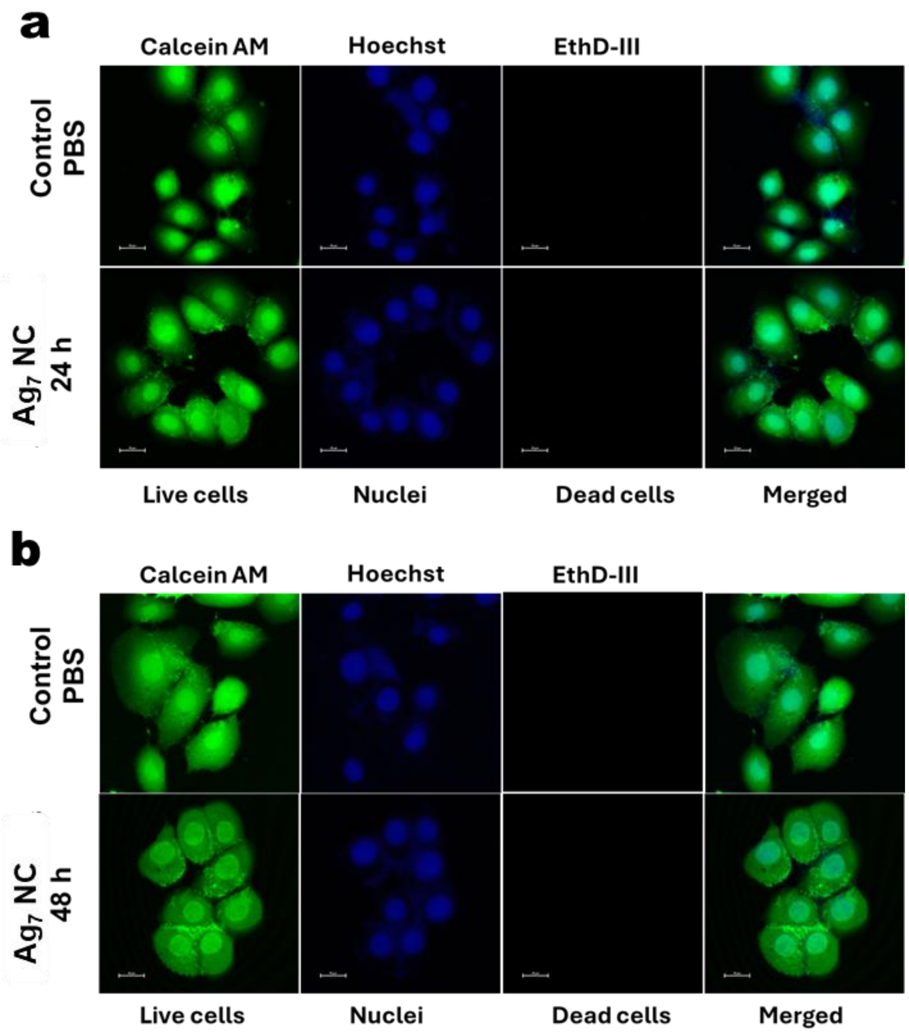

Figure S10. Live/dead cell staining of MCF-10A normal cells treated with Ag_7 NCs. Representative fluorescence images of the distribution of MCF-10A live and dead cells untreated and treated with Ag_7 NCs for a) 24 and b) 48h. The nuclei, live cells and dead cells were stained by Hoechst 33342 (blue), calcein AM (green) and EthD-III (red), respectively. Scale bars, 20 µm.

Figure S11

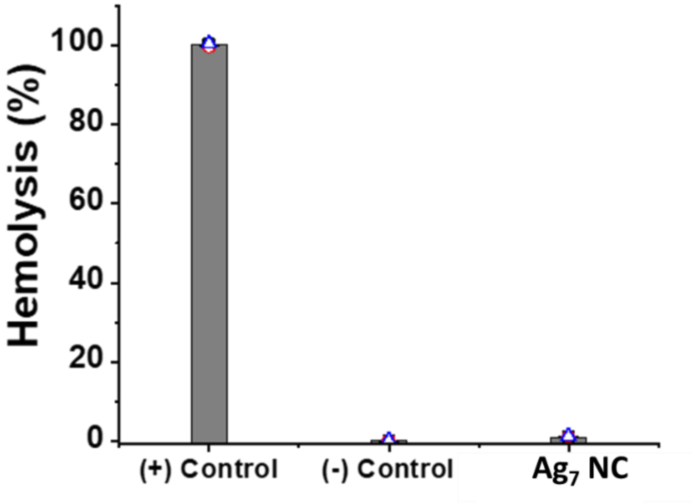

Figure S11. Hemolysis assessment of Ag_7 NCs. Percent hemolysis of sheep red blood cells (RBCs) after incubation with Ag_7, calculated from OD_{540nm} using PBS as the negative control (0% hemolysis) and deionized water as the positive control (100% hemolysis). To avoid absorbance overlap from the Ag_7 NCs, the OD_{540nm} values of Ag_7-treated samples were background-corrected by subtracting the OD_{540nm} of Ag_7 NC dispersed in PBS without RBCs. Data are presented as mean ± s.e.m (n = 3 independent replicates), demonstrating negligible hemolysis for Ag_7 under the tested conditions.

Figure S12

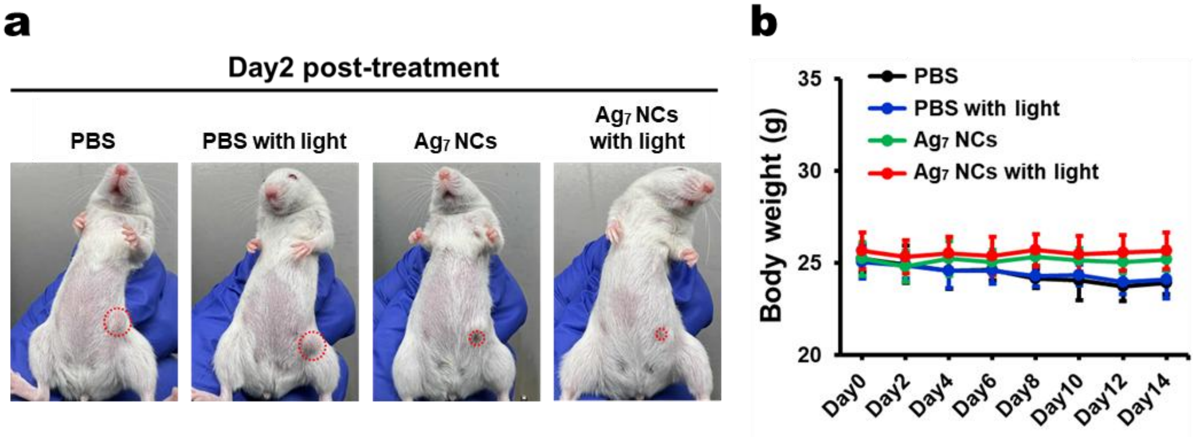

Figure S12. *In vivo* evaluation of Ag_7 NC treatment. (a) Tumor morphology after 2 days post-treatment. The red dashed circles indicate the tumor regions. (b) Body weight of experimental mice in each group.

Figure S13

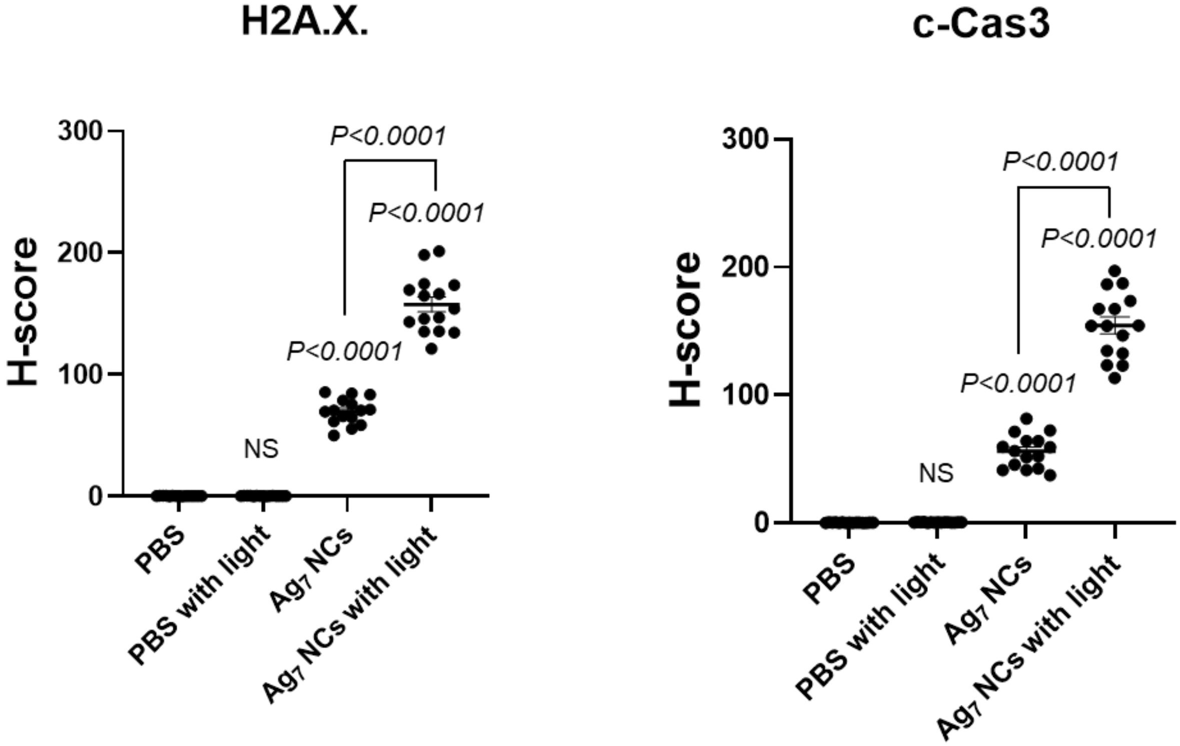

Figure S13. H-score analysis of H2A.X. and c-Cas3 from Figure 5e. For each mouse, three random fields of view were selected for imaging and quantification, yielding a total of 15 measurements per group. H-scores were defined and quantified using QuPath software. Data are presented as mean ± SD, with p-values calculated using One-way ANOVA followed by Tukey's multiple comparisons test.

Figure S14

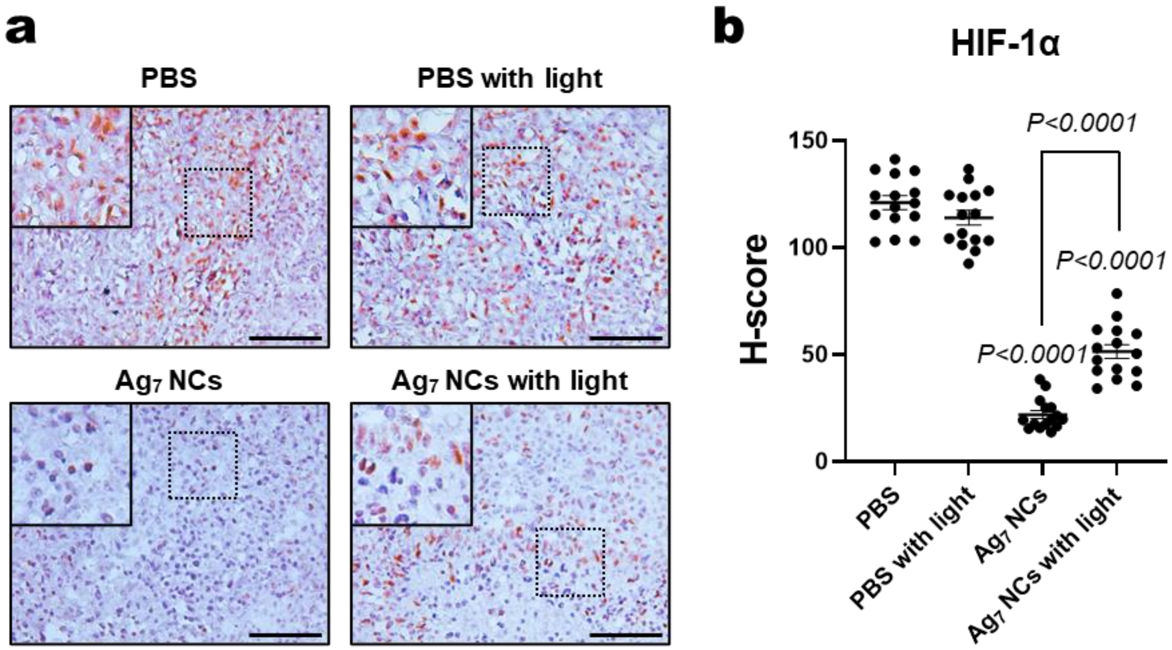

Figure S14. Validation of intratumoral hypoxia relief and self-oxygenated PDT. (a) Expression of HIF-1 alpha (D1S7W) assessed by IHC staining (scale bar, 100 μm). Dashed boxes indicate zoomed-in regions. Experiments were independently repeated at least three times with consistent results, and one representative experiment is shown. (b) H-scores were defined and quantified using QuPath software. For each mouse, three random fields of view were selected for imaging and quantification, yielding a total of 15 measurements per group. Data are presented as mean ± SD, with p-values calculated using One-way ANOVA followed by Tukey's multiple comparisons test.

Figure S15

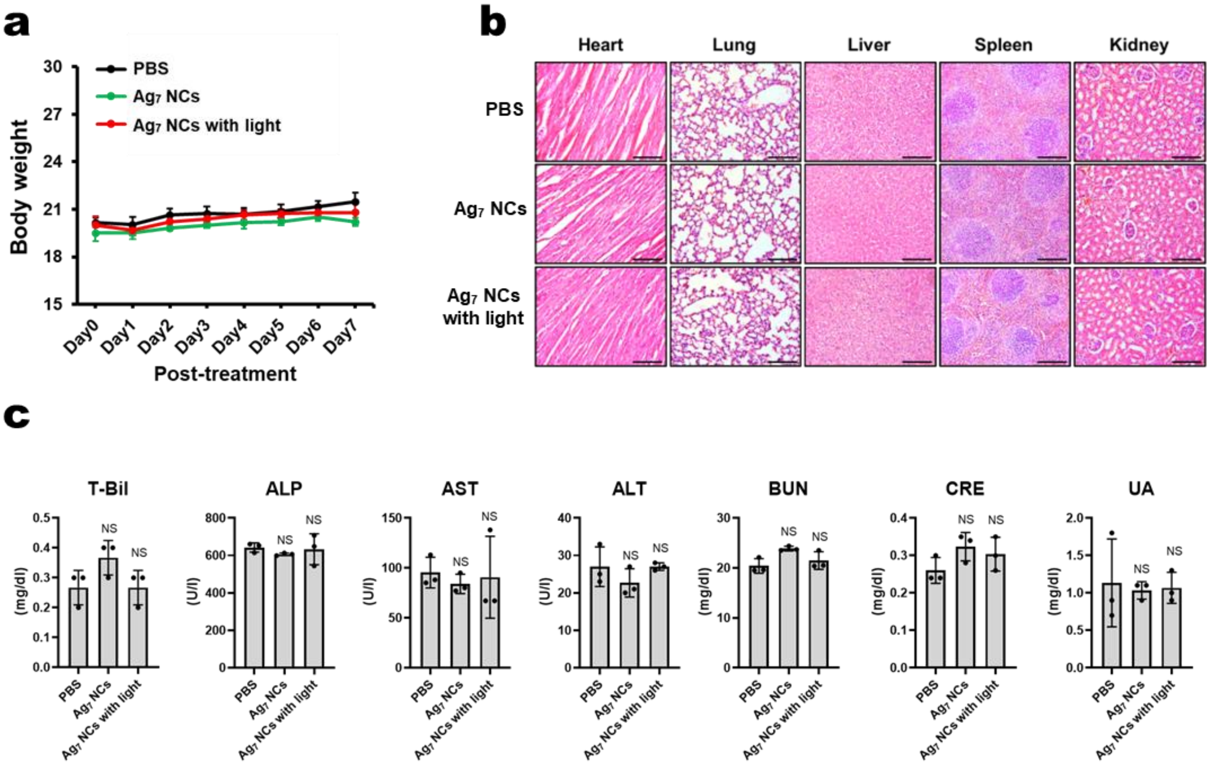

d

	GLU (mg/dL)	PRO (mg/dL)	ALB (mg/dL)	BIL (mg/dL)	CRE (mg/dL)	PH	BLD (mg/dL)	KET (mg/dL)	NIT	LEU (Leu/ul)
	-	-	1	-	50	6.5	-	-	-	-
PBS	-	-	1	-	50	6.5	-	-	-	-
	-	-	1	-	50	6.5	-	-	-	-
	-	-	5	-	50	6.5	-	-	-	-
Ag_7 NCs	-	-	1	-	50	6.5	-	-	-	-
	-	-	5	-	50	6.5	-	-	-	-
	-	-	5	-	50	6.5	-	-	-	-
Ag_7 NCs with light	-	-	1	-	50	6.5	-	-	-	-
	-	-	5	-	50	6.5	-	-	-	-

Figure S15. *In vivo* biosafety of Ag_7 NCs. BALB/c mice received an intramammary injection of 300 ppm Ag_7 NCs, followed by irradiation with a 730 nm NIR laser (71 mW/cm^2) for 20 min, at 24 h post-injection. All treatments were administered as a single dose. (a) Body weight of experimental mice in each group (n = 3). (b) Histological morphology of the heart, lung, liver,

spleen, and kidney in each group after 7 days post-treatment was assessed by H&E staining (scale bar: 100 μm). Experiments were independently repeated at least three times with consistent results, and one representative experiment is shown. (c) Serum and (d) urine biochemical parameters in each group after 7 days post-treatment. Data are presented as mean ± SD, with p-values calculated using One-way ANOVA followed by Tukey's multiple comparisons test.
